# Chemical Variation among Castes, Female Life Stages and Populations of the Facultative Eusocial Sweat Bee *Halictus rubicundus* (Hymenoptera: Halictidae)

**DOI:** 10.1007/s10886-021-01267-w

**Published:** 2021-03-31

**Authors:** Iris Steitz, Robert J Paxton, Stefan Schulz, Manfred Ayasse

**Affiliations:** 1 Institute of Evolutionary Ecology and Conservation Genomics, University of Ulm, Ulm, Germany; 2 General Zoology, Institute for Biology, Martin Luther University Halle-Wittenberg, Halle (Saale), Germany; 3 German Centre for Integrative Biodiversity Research (iDiv) Halle-Jena-Leipzig, Leipzig, Germany; 4 Departement of Life Sciences, Institute of Organic Chemistry, TU Braunschweig, Braunschweig, Germany

**Keywords:** Halictid bee, Facultative eusocial behavior, Chemical communication, Regulation of reproduction, Population dialect

## Abstract

In eusocial insects, chemical communication is crucial for mediating many aspects of social activities, especially the regulation of reproduction. Though queen signals are known to decrease ovarian activation of workers in highly eusocial species, little is known about their evolution. In contrast, some primitively eusocial species are thought to control worker reproduction through physical aggression by the queen rather than via pheromones, suggesting the evolutionary establishment of chemical signals with more derived sociality. However, studies supporting this hypothesis are largely missing. Socially polymorphic halictid bees, such as *Halictus rubicundus*, with social and solitary populations in both Europe and North America, offer excellent opportunities to illuminate the evolution of caste-specific signals. Here we compared the chemical profiles of social and solitary populations from both continents and tested whether (i) population or social level affect chemical dissimilarity and whether (ii) caste-specific patterns reflect a conserved queen signal. Our results demonstrate unique odor profiles of European and North American populations, mainly due to different isomers of n-alkenes and macrocyclic lactones; chemical differences may be indicative of phylogeographic drift in odor profiles. We also found common compounds overproduced in queens compared to workers in both populations, indicating a potential conserved queen signal. However, North American populations have a lower caste-specific chemical dissimilarity than European populations which raises the question if both use different mechanisms of regulating reproductive division of labor. Therefore, our study gives new insights into the evolution of eusocial behavior and the role of chemical communication in the inhibition of reproduction.

**Supplementary Information:**

The online version contains supplementary material available at 10.1007/s10886-021-01267-w.

## Introduction

Chemical communication plays a crucial role in insects, especially in taxa that live in social groups. Chemical signals are known to regulate many aspects of their social activities, including the mediation of various types of inter- and intracolonial recognition (Richard and Hunt 2013; Wilson 1965). It is thought that the complexity of chemical communication and information transfer increases across species with the complexity of their social organization (Leonhardt et al. 2016; Steitz et al. 2018). Insects comprise a huge variety of social organizations and interactions ranging from solitary species, were contact among conspecifics is very rare and limited to interactions of mates, to advanced eusocial species with a marked reproductive division of labor, in which every individual performs its specific task to the benefit of the colony (Wilson 1971).

In eusocial insects, chemical communication is known to be involved not only in nestmate and kin recognition but also in regulating reproductive division of labor and task allocation of individuals in the society (Ayasse et al. 2001; Kocher and Grozinger 2011; Rottler-Hoermann et al. 2016; Steitz and Ayasse 2020; Steitz et al. 2018, 2019). The castes, namely the reproductive queens and the non-reproductive workers, often differ in their chemical signatures and signals produced by queens or dominant females are thought to regulate many aspects of social behavior and reproduction within the colony (Le Conte and Hefetz 2008; Soro et al. 2011; Sramkova et al. 2008; Steitz and Ayasse 2020; Steitz et al. 2018, 2019; Oi et al. 2015). These chemical queen signals are known to decrease the ovarian activity of workers and have been the focus of much attention in various eusocial insect species, especially those with highly developed eusocial behavior (Ayasse and Jarau 2014; Holman et al. 2013; Holman et al. 2016; Monnin 2006; Oi et al. 2015; Smith et al. 2012, 2016; Sramkova et al. 2008; Van Oystaeyen et al. 2014). Despite this, in primitively eusocial insect species with a lack of morphologically distinct castes, it is thought that queens may regulate the reproduction of workers through physical aggression rather than through chemical signals (Bourke 1999; Oi et al. 2019; Smith and Liebig 2017). Maintenance of reproductive division of labor through queen behavior and not chemical signature has, for example, already been shown for *Polistes* wasps (Oi et al. 2019) and bumble bee species (Amsalem et al. 2017; Padilla et al. 2016; Starkey et al. 2019). However, a more recent study on a primitively eusocial sweat bee, *Lasioglossum malachurum*, revealed that queen chemical signals are indeed sufficient to decrease worker ovarian activity without any physical contact between the castes, indicating a more complex evolutionary picture of reproductive division of labor (Steitz and Ayasse 2020). Overall, little is known about the evolution of reproductive division of labor and caste-specific signals, in large part due to past focus on species with highly developed eusocial behavior rather than species with a more flexible social behavior. Indeed, these species with a higher flexibility of eusocial behavior might better represent the transition from solitary living to eusocial behavior and are therefore highly useful to get insights on this transition.

Halictid bees or sweat bees are ideal organisms to study the evolution of caste-specific traits as (i) they evolved eusociality much more recently than the advanced eusocial taxa such as the ants, honey bees and vespid wasps (Brady et al. 2006; Gibbs et al. 2012; Kocher and Paxton 2014), (ii) there was more than one independent origin of eusociality in the taxon (Brady et al. 2006; Gibbs et al. 2012; Greenberg 1979), (iii) they possess several reversals from eusociality back to solitary nesting (Danforth 2002; Danforth et al. 2003; Wcislo and Danforth 1997) and (iv) they show considerable diversity in social behavior, ranging from solitary to primitively eusocial species, and including communal and socially polymorphic forms (Michener 1974; Yanega 1989). Especially the socially polymorphic species, in which one species can express either social or solitary behavior, are suitable model organisms to study the evolution of caste-specific odor traits (e.g. queen signals) as they enable comparison among social and solitary behavioral forms in a potentially common genetic background (Kocher and Paxton 2014; Shell and Rehan 2018).

One especially interesting species is *Halictus rubicundus*, an ancestrally eusocial halictine species with a Holarctic distribution which exhibits social polymorphism, with both solitary and social nests in both North America and Europe (Danforth 2002; Danforth et al. 1999; Eickwort et al. 1996; Soucy 2002; Soucy and Danforth 2002; Yanega 1989; Field et al. 2010). Social living comprising at least two broods per year, one worker brood and one brood containing the sexuals, occurs in regions with longer growing seasons, e.g. in lower elevations, as in New York (Yanega 1989) or the Netherlands (Hogendoorn and Leys 1997). In cooler regions, e.g. at higher altitude or latitude as in the Rocky Mountains, Colorado (Eickwort et al. 1996) or in Scotland (Potts and Willmer 1997; Field et al. 2010), *H. rubicundus* is solitary and produces only one brood, containing the sexuals, per year.

A phylogeographic study of *H. rubicundus* based on mitochondrial DNA revealed that, in North America, eusocial populations clustered into one lineage and solitary populations clustered into a second lineage, independent of their geographic locality (Soucy and Danforth 2002), providing evidence for a genetic basis to their social behavior. This is reinforced by size differences; in North American social populations, queens and workers exhibit marked size variation whereas in North American solitary populations, individuals are of intermediary size (Soucy 2002). In Europe, in contrast, Soucy and Danforth (2002) showed that social and solitary populations all clustered together in a third lineage distant to the two North American lineages, suggesting considerable genetic differentiation between all North American and all European populations. Moreover, in Great Britain, *H. rubicundus* was shown to exhibit social plasticity; females collected from social nests could switch to solitary living when they were relocated to regions where *H. rubicundus* is natively solitary and *vice versa* (Field et al. 2010; Field et al. 2012). Social plasticity is also represented by body size differences, as originally solitary females, transplanted to regions where females are social, produced offspring of the same size as native social females (Field et al. 2012), though caste differences in size in Europe were modest in comparison to North American eusocial populations. Though Soro et al. (2010) have shown subtle genetic differentiation between eusocial and solitary population of European *H. rubicundus* using nuclear (microsatellite) markers, the common garden experiments of Field et al. (2010) indicate that social phenotype is to some extent plastic in sweat bees in response to local environmental conditions (Field et al. 2010, 2012).

Although, sweat bees and especially *H. rubicundus* are ideal organisms to study traits linked to the evolution of social behavior, including their chemical communication, few studies have addressed their chemical profiles or the evolution of caste-specific or queen signals. The chemical profile of one obligate eusocial halictid species, *L. malachurum*, is well characterized; it consists of compounds mainly belonging to the substance classes of n-alkanes, n-alkenes, macrocyclic lactones, isopentenyl esters of unsaturated fatty acids, and ethyl esters (Ayasse et al. 1993; Ayasse et al. 1999; Soro et al. 2011; Steitz et al. 2018; Steitz and Ayasse 2020). Whereas isopentenyl esters of unsaturated fatty acids play a key role in the female sex pheromone (Ayasse et al. 1999), macrocyclic lactones have been shown to mediate aggressive interactions between nest-founding queens (gynes; Smith and Weller 1989). Moreover, n-alkanes and macrocyclic lactones have been found in larger quantities in breeding queens compared with workers in *L. malachurum* (Ayasse et al. 1993, 1999; Steitz et al. 2018; Steitz and Ayasse 2020), though macrocyclic lactones are sufficient to influence worker behavior, distracting workers from activating their ovaries and therefore functioning as a queen signal in this species (Steitz and Ayasse 2020). Indeed, macrocyclic lactones are the single compound class which is at higher titer in queens compared to workers in several eusocial halictid bee species, suggesting that it may function as a queen pheromone among several species in this taxon (Steitz et al. 2018, 2019). Additionally, queens and workers are chemically more distinct in obligate eusocial compared to socially polymorphic species like *H. rubicundus*, indicating a more complex caste-specific chemical profile with a more complex degree of social organization in halictid bees (Steitz et al. 2018). Population-specific differences and direct comparison among social and solitary populations of *H. rubicundus* may shed further light on the evolution of caste-specific signals and their mode of regulating worker reproduction in sweat bees.

The aim of our study was to investigate early stages of caste differentiation by examining the chemical profiles of different castes and female life stages among *H. rubicundus* females from social and solitary populations in Europe and North America. We hypothesized that the chemical profiles of European and North American populations would be distinct due to geographic isolation and odor drift. Overlying these continental differences, we hypothesized patterns of potential queen signals in the social populations common to Europe and North America due to the same or similar evolutionary pathways to the regulation of worker reproduction. We complemented these analyses by measuring body sizes of different female groups of social and solitary European and North American females as a metric of caste.

## Material and Methods

### Bee Collection

**Fig. 1:**
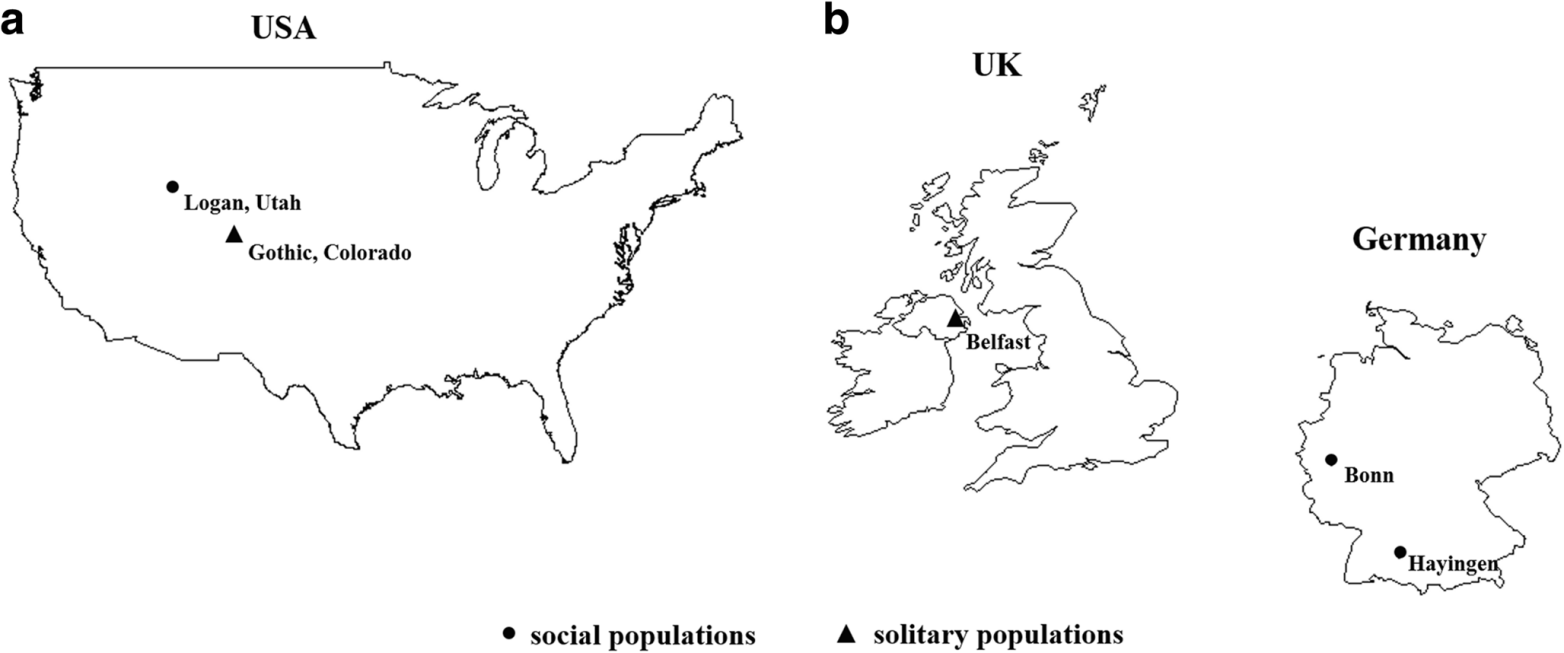
Collection sites of *H. rubicundus* females from social (dot) and solitary (triangle) populations of (**a**) North America and (**b**) Europe

*Halictus rubicundus* females at various life stages were collected from sites in Europe and North America: 1) solitary nest foundresses (*n* = 20) and solitary breeding females (n = 20) in spring and summer 2011 in Belfast, Northern Ireland; 2) nest foundresses (*n* = 21), social breeding queens (*n* = 22) and workers (*n* = 47) of social populations in spring and summer 2013 in Bonn and Hayingen, Germany; 3) solitary breeding females (*n* = 14) in summer 2016 in Gothic, Colorado, USA; and 4) breeding queens (*n* = 11) and workers (*n* = 37) of a social population in summer 2016 in Logan, Utah, USA (Fig. 1). We collected all females at their nest sites by using an insect net or a vacuum suction device as described in Soro et al. (2009). The bees were placed in individual, small plastic vials (Eppendorf tubes: 1.5 ml) and killed by freezing at −40 °C for further use in chemical analyses. As chemical compounds, especially cuticular hydrocarbons are known to exhibit a long-term stability (Guillem et al. 2016; Martin et al. 2009), we exclude that the collection of bees in different years may have any impact on our results.

### Chemical Analyses

Frozen females were individually rinsed for 15 s in 500 μl n-pentane (Uvasol, 99.5%, Merck, Germany) to extract cuticle surface compounds. All extracts were concentrated under a gentle stream of nitrogen to 25% of the original volume. For quantitative analysis, 10 μl n-octadecane (stock solution: 100 μg/ml in n-hexane) were added to each extract as an internal standard.

Chemical analyses were performed on an Agilent 7820 A Series gas chromatograph (Agilent Technologies, Germany) equipped with a non-polar DB-5 MS capillary column (30 m × 0.25 mm inner diameter, J&W) and a flame ionization detector using hydrogen as a carrier gas (constant flow, 2.0 ml/min). One microliter of each sample was injected splitless into the gas chromatograph (injector temperature: 310 °C), operating at 50 °C for 1 min, after which the split valve was opened, and the temperature was increased continuously by 10 °C/min to a final temperature of 310 °C. The structural elucidation of individual compounds was based on gas chromatography/mass spectrometry (HP 6890 series, Hewlett-Packard, Germany; method as described above for GC, carrier gas: helium) and on the comparisons of mass spectra using references from the NIST11 library and GC retention times with those of authentic reference samples by using AMDIS 2.71 (Automated Mass Spectral Deconvolution and Identification System). The absolute amounts of all substances were determined by using Agilent ChemStation Software (Agilent Technologies, Germany) and the internal standard as a reference. In order to estimate relative proportions for further downstream analyses, absolute amounts of individual compounds were divided by the sum of the absolute amounts of all compounds and multiplied by 100.

To identify the double bond positions in n-alkenes and macrocyclic lactones, we separated pools of extracts into polar (macrocyclic lactones) and unpolar (n-alkenes) fractions using the SPE-method (solid-phase extraction) according to Naß et al. (1998). The double bond positions were identified by derivatizing with dimethyl disulfide (DMDS) according to Carlson et al. (1989).

### Measurement of Physiological State and Size

After extraction of cuticle surfaces, all bees were dissected under a stereomicroscope to check ovarian stage, which was classified into five categories according to Duchateau and Velthuis (1989) so as to the physiological state and caste of each female. Head width (maximal width of the head including the compound eyes) was used as a proxy of size and was measured for each female using an eyepiece graticule.

### Statistics

Cuticular odor bouquets of *H. rubicundus* females from various life stages (social nest foundresses, social breeding queens, workers, solitary nest foundresses and solitary breeding females) were analyzed to test for differences among female groups, social organization (social vs. solitary) or population sites (Europe vs. North America). Relative amounts [%] of each compound were calculated with respect to the total concentration of the whole bouquet; peaks with a concentration < 0.01% were excluded from downstream analyses.

To visualize dissimilarities between groups, we performed non-metric multidimensional scaling (NMDS) based on the Bray-Curtis dissimilarities, as implemented in Primer (Clarke and Gorley 2006). Furthermore, we performed one-way ANOSIM (analysis of similarities, permutations: 10,000) following post-hoc SIMPER tests to check for relative contributions of different compounds to detected differences between groups. To check for caste-specifically expressed compounds potentially involved in regulating the reproductive division of labor, we compared the expression level of each compound between breeding queens and workers from European and North American populations by performing univariate Mann-Whitney-U tests with Benjamini-Hochberg correction.

To compare chemical dissimilarities among female groups, social organization or population sites, we calculated Bray-Curtis dissimilarities based on the square-root-transformed cuticle odor bouquet datasets (relative proportions) using the *vegan* package (Oksanen et al. 2017) of R v. 3.3.1 (R Core Team 2016). The obtained pairwise dissimilarity values were compared by using generalized linear models with a quasi-Poisson error distribution followed by a Tukey post-hoc test for pairwise comparisons using the R packages *stats* and *multcomp* (Hothorn et al. 2008).

To check for size differences among females of different groups, population sites or social organization, we compared their head widths using linear models with log-transformed data followed by a Tukey post-hoc test for pairwise comparisons.

## Results

### Odor Profiles of Female Life Stages and Populations

**Fig. 2:**
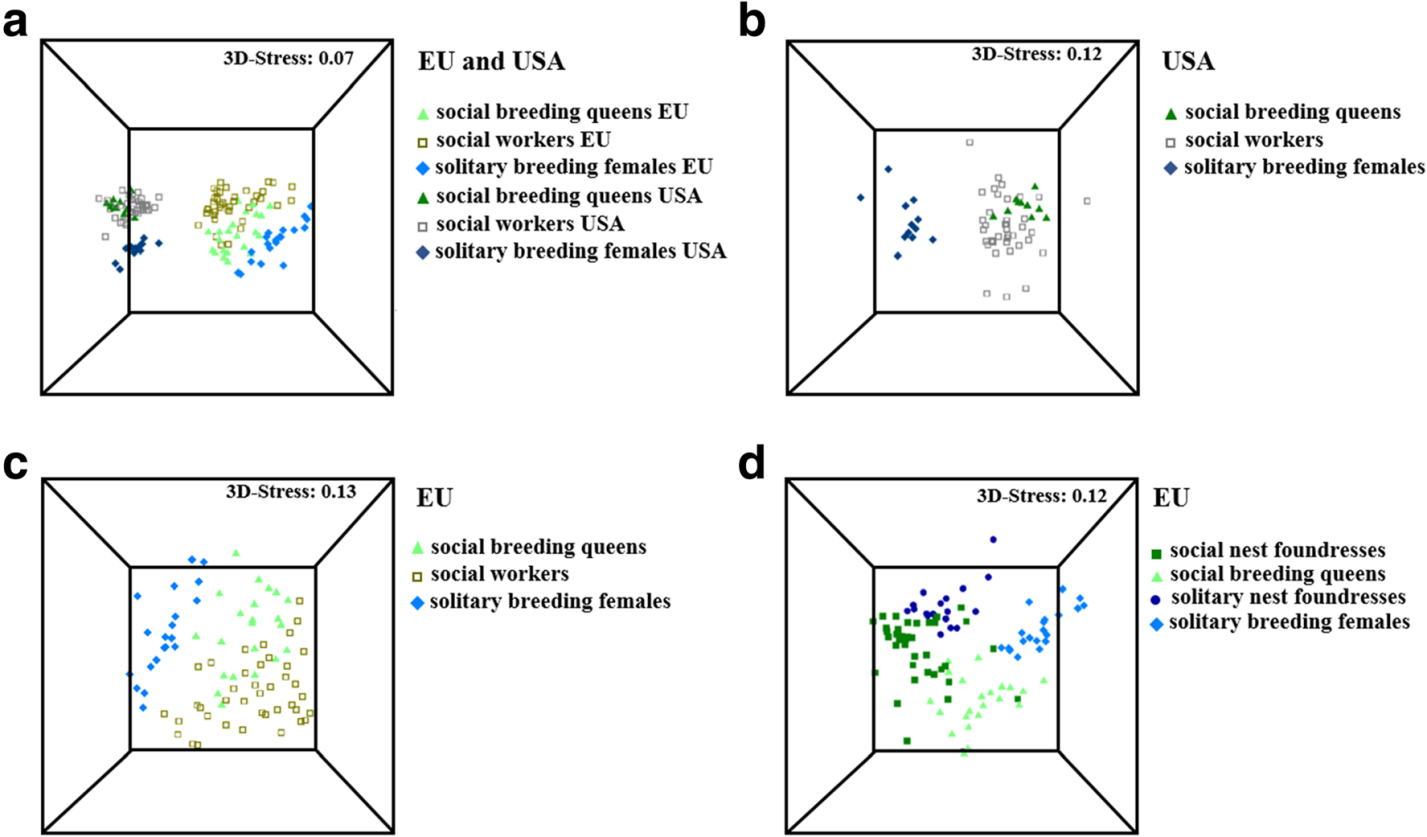
Differences in cuticular chemical profiles among female groups based on relative amounts of compounds. In total, we compared (**a**) social (EU: *n* = 22; USA: *n* = 11) and solitary (EU: *n* = 20; USA: *n* = 14) breeding females and workers (EU: *n* = 47; USA: *n* = 37) between North American and European populations (*NMDS*, Bray-Curtis similarity measures, 3D-Stress: 0.07), **b** social (*n* = 11) and solitary (*n* = 14) breeding females and workers from North America (*NMDS*, Bray-Curtis similarity measures, 3D-Stress: 0.12), **c** social (*n* = 22) and solitary (*n* = 20) breeding females and workers (*n* = 47) from the European populations (*NMDS*, Bray-Curtis similarity measures, 3D-Stress: 0.13) and **d** social and solitary nest foundresses (social: *n* = 21; solitary: n = 20) and breeding females (social: n = 22; solitary: n = 20) from the European populations (*NMDS*, Bray-Curtis similarity measures, 3D-Stress: 0.12). Stress-values represent the accuracy of the data representation in reduced dimensions. Stress-values <0.2 provide a good representation in the given number of dimensions

In total, we found 158 different chemical compounds among all tested *H. rubicundus* female groups. Overall, 52 of these compounds were exclusively found in females from North America, whereas 56 compounds were only found in the European females, including n-alkenes and macrocyclic lactones with different double bond positions (Fig. S1, Table S1). We found a clear separation of European females from North American females within each group (*ANOSIM*, *global R* = 0.801*, P < 0.001*, all pairwise comparisons between European and North American females: *P* < 0.001; Fig. 2a, Table S2). The majority (92, 6%) of the identified compounds belong to six different compound classes: n-alkanes, n-alkenes, saturated and unsaturated macrocyclic lactones, fatty acids, ethyl esters and isopentenyl esters (Table S1).

**Fig. 3:**
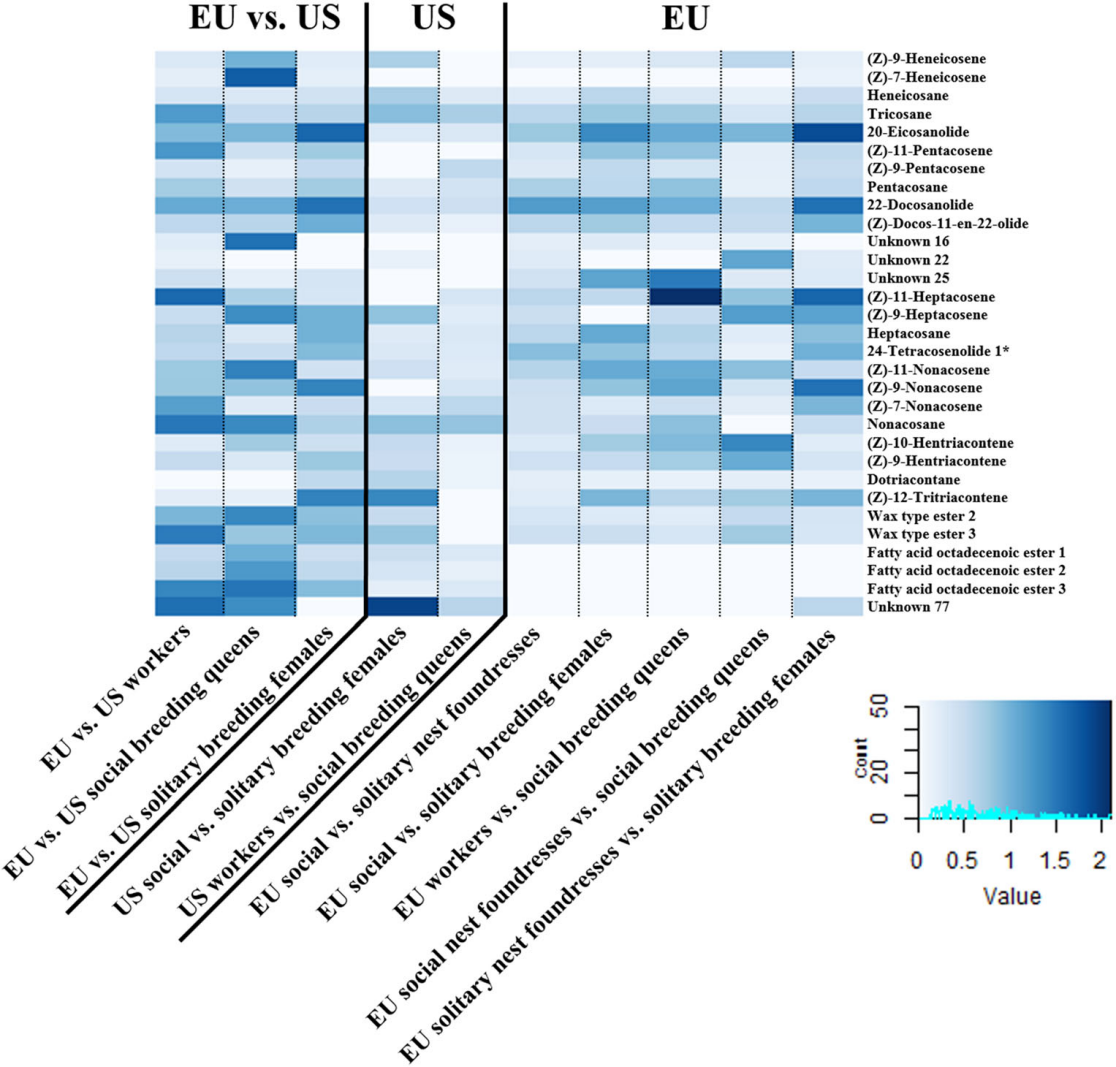
Heatmap of Bray-Curtis dissimilarities of those compounds which contributed most to the chemical differences of all tested female groups

Regarding only North America, social and solitary groups were chemically distinct (*ANOSIM*, *global R* = 0.512, *P* < 0.001, all pairwise comparisons between solitary and social female groups: *P* < 0.001; Fig. 2b), whereas queens and workers were chemically not distinct (*ANOSIM*, *global R* = 0.512, *P* < 0.001, pairwise comparison between queens and workers: *P* = 0.269; Fig. 2b). Chemical dissimilarities between social and solitary females were mainly caused by different relative amounts of the n-alkanes heneicosane, tricosane, nonacosane and dotriacontane, the n-alkenes (*Z*)-9-pentacosene, (*Z*)-11-heptacosene, (*Z*)-9-heptacosene and (*Z*)-7-nonacosene as well as three unknown compounds (*SIMPER* analyses, each compound contributed more than 2.0% to total Bray-Curtis dissimilarity; Fig. 3, Table S2).

Concerning only European populations, we also found distinct chemical profiles between social and solitary groups as well as between queens and workers within the social populations (*ANOSIM*, *global R* = 0.521, *P* < 0.001, all pairwise comparisons: *P* < 0.001; Fig. 2c). Chemical differences between social and solitary groups were mainly due to the relative amounts of the n-alkanes tricosane, pentacosane and heptacosane, the n-alkenes (*Z*)-11-pentacosene, (*Z*)-11-heptacosene, (*Z*)-11-nonacosene, (*Z*)-9-nonacosene, (*Z*)-10-hentriacontene and (*Z*)-12-tritriacontene as well as the saturated macrocyclic lactones 20-eicosanolide and 22-docosanolide and the unsaturated macrocyclic lactones (*Z*)-docos-11-en-22-olide and (*Z*)-tetracos-11-en-24-olide (*SIMPER* analyses, each compound contributed more than 2.0% to total Bray-Curtis dissimilarity; Fig. 3, Table S2). European social breeding queens and workers were mainly chemically distinct due to the relative amounts of the n-alkanes tricosane, pentacosane and nonacosane, the n-alkenes (*Z*)-11-pentacosene, (*Z*)-11-heptacosene, (*Z*)-11-nonacosene, (*Z*)-9-nonacosene, (*Z*)-10-hentriacontene and (*Z*)-9-hentriacontene as well as the saturated macrocyclic lactones 20-eicosanolide and 22-docosanolide (SIMPER analyses, each compound contributed more than 2.0% to total Bray-Curtis dissimilarity; Fig. 3, Table S2).

We also found clear chemical distinctions among nest founding females and breeding females, both among social and among solitary nesting bees from Europe (*ANOSIM*, *global R* = 0.544, *P* < 0.001, all pairwise comparisons: *P* < 0.001; Fig. 2d). Differences between nest foundresses and breeding queens in social populations were mainly due to the relative amounts of the n-alkanes tricosane and nonacosane, the n-alkenes (*Z*)-11-pentacosene, (*Z*)-11-heptacosene, (*Z*)-9-heptacosene, (*Z*)-11-nonacosene, (*Z*)-9-nonacosene, (*Z*)-7-nonacosene and (*Z*)-10-hentriacontene and the saturated macrocyclic lactones 20-eicosanolide and 22-docosanolide (*SIMPER* analyses, each compound contributed more than 2.0% to total Bray-Curtis dissimilarity; Fig. 3, Table S2). In comparison, nest foundresses and breeding females of solitary populations mainly differed due to the relative amounts of the n-alkane heptacosane, the n-alkenes (*Z*)-11-heptacosene, (*Z*)-9-heptacosene, (*Z*)-9-nonacosene, (*Z*)-7-nonacosene, (*Z*)-12-tritriacontene, the saturated macrocyclic lactones 20-eicosanolide and 22-docosanolide as well as the unsaturated macrocyclic lactones (*Z*)-docos-11-en-22-olide and (*Z*)-tetracos-11-en-24-olide (*SIMPER* analyses, each compound contributed more than 2.0% to total Bray-Curtis dissimilarity; Fig. 3, Table S2).

### Chemical Dissimilarities among Various Female Life Stages and Populations

**Fig. 4:**
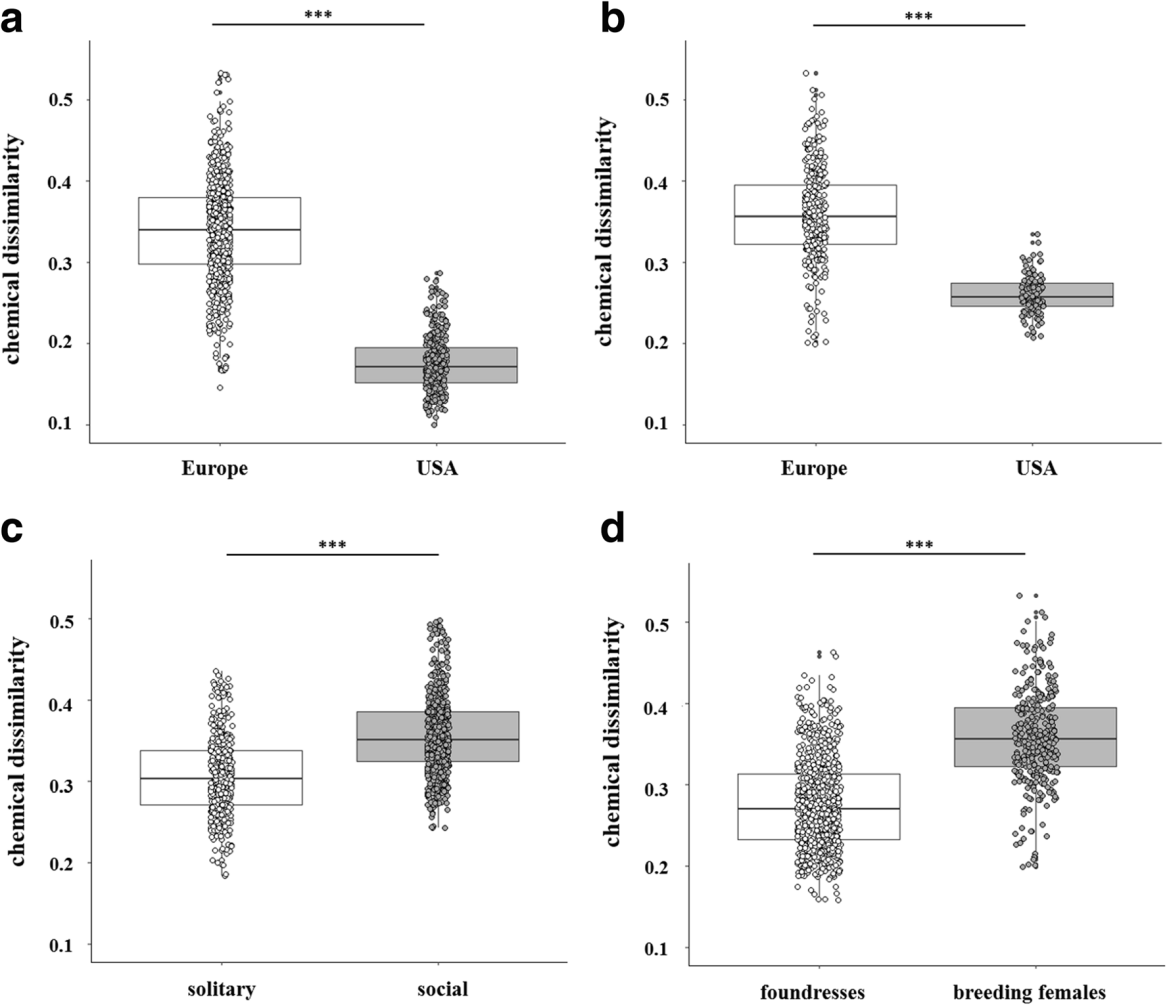
Comparison of chemical distances (Bray-Curtis dissimilarities) between (**a**) queens (EU: n = 22; USA: n = 11) and workers (EU: n = 47; USA: *n* = 37) of social populations in Europe and North America, **b** social (EU: n = 22; USA: n = 11) and solitary (EU: n = 20; USA: n = 14) breeding females of populations in Europe and North America, **c** nest foundresses (solitary: n = 20; social: *n* = 21) and breeding queens (solitary: n = 20; social: n = 22) in European solitary and social populations and **d** social (foundresses: n = 21; breeding females: n = 22) and solitary populations (foundresses and breeding females: n = 20), comparing foundresses or breeding females of European populations. European populations exhibited higher chemical dissimilarities between castes (**a**; *quasi-Poisson GLM*, *P* < 0.001) and social and solitary breeding females (**b**; *quasi-Poisson GLM*, *P* < 0.001). Beside this, chemical distinction was higher between nest foundress and breeding queens in social than in solitary European populations (**c**; *quasi-Poisson GLM*, *P* < 0.001), whereas chemical dissimilarities were higher in breeding queens between social and solitary populations than in nest foundresses (**d**; *quasi-Poisson GLM*, *P* < 0.001). Boxes represent the median and 25th and 75th percentile, and the overlaying dots represent all calculated data values

**Fig. 5:**
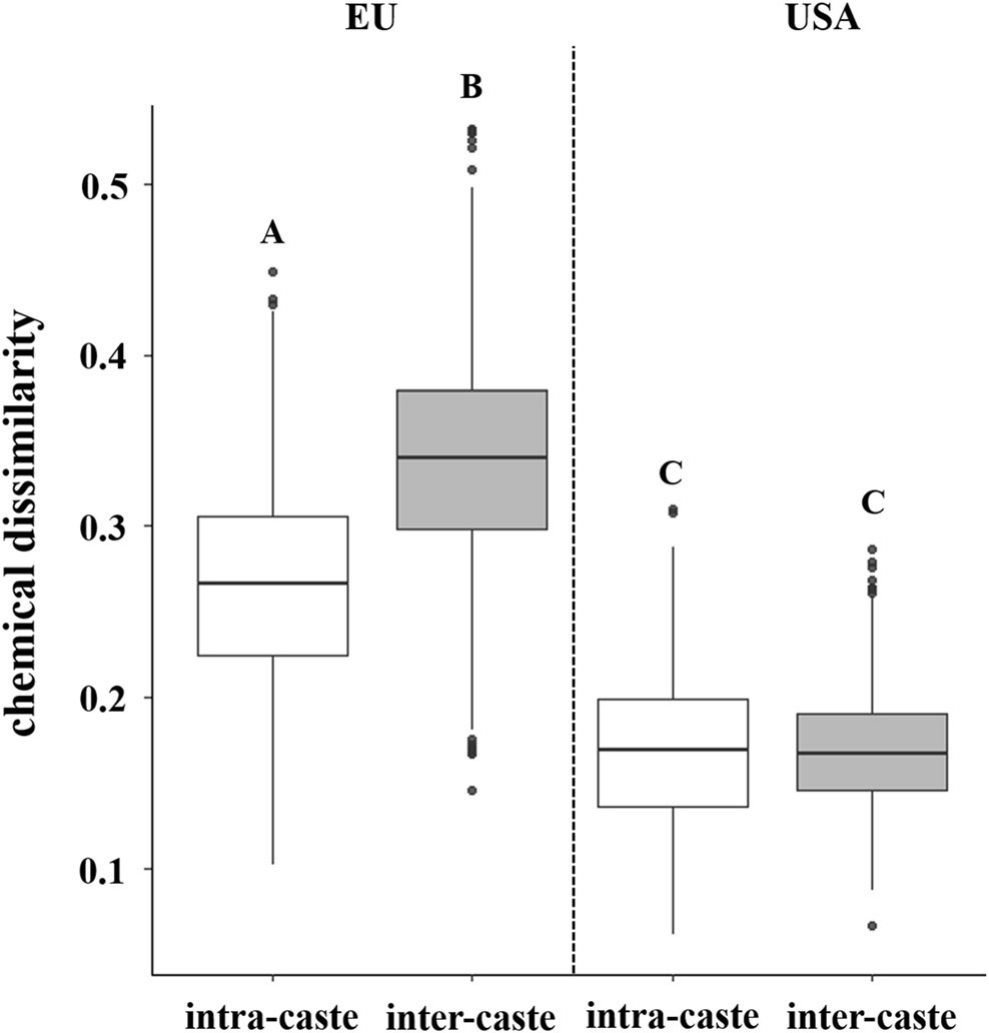
Comparison of chemical dissimilarities within castes (intra-caste, white) and between castes (inter-caste, grey) of social populations from Europe (EU) and North America (USA). In European populations, intra-caste chemical dissimilarity was significantly lower than inter-caste chemical dissimilarity (*quasi-Poisson GLM*, *P* < 0.001). Regarding North American females, intra-caste and inter-caste chemical dissimilarities did not differ significantly (*quasi-Poisson GLM*, *P* = 0.997). Boxes represent the median and 25th and 75th percentile. Different letters indicate significant differences. Different letters indicate significant differences between the groups

We found higher chemical dissimilarities in chemical profiles between breeding queens and workers in the social nests from Europe compared to the social nests from North America (*quasi-Poisson GLM*, *F*_*1,1018*_ = 2425.3, *P* < 0.001; Fig. 4a). Despite this, we could not find any difference between comparisons among nestmates or non-nestmates in this queen-worker comparison (*quasi-Poisson GLM*, *F*_*1,1018*_ = 34,732, *P* = 0.775). In addition, we found higher chemical distinction between social and solitary breeding females in the European populations compared to those from North America (*quasi-Poisson GLM*, *F*_*1,438*_ = 351.77, *P* < 0.001; Fig. 4b). As we also collected nest founding females in Europe, we tested whether the dissimilarities in their chemical profiles was different for social and solitary populations. We found higher chemical distinction between nest foundresses and breeding females in social European populations compared to solitary populations (*quasi-Poisson GLM*, *F*_*1,1006*_ = 230.3, *P* < 0.001; Fig. 4c). In addition, we checked whether nest founding females of social and solitary populations are chemically less distinct than breeding females of both populations or whether we could find similar patterns in both groups when females initiate breeding. We found higher chemical distinction in breeding females of social and solitary populations compared to nest foundresses (*quasi-Poisson GLM*, *F*_*1,1078*_ = 449.17, *P* < 0.001; Fig. 4d). Despite this, the inter-caste chemical dissimilarity of queens and workers from European populations was significantly higher than the intra-caste dissimilarity (*quasi-Poisson GLM*, *F*_*1,2002*_ = 1392.1, *P* < 0.001; Fig. 5). This result was not true for North American populations, in which intra- and inter-caste chemical dissimilarity was not significantly different (*quasi-Poisson GLM*, *F*_*1,2002*_ = 1392.1, *P* = 0.997; Fig. 5).

### Caste Differences of Single Chemical Compounds

In univariate tests, we found caste-specific differences in the single amounts of chemical compounds between breeding queens and workers in both European (13 compounds) and North American (13 compounds) populations (*Mann-Whitney-U tests with Benjamini-Hochberg correction*, *adjusted P* < 0.05; Table 1) mainly belonging to the compound classes of n-alkanes, n-alkenes, methyl-alkanes and isopentenyl esters. However, there were only 3 compounds which showed a caste difference in both tested populations, whereas the majority of the compounds showed a caste-specific expression in either Europe or North America. Those compounds, which exhibited higher amounts in queens compared to workers in both populations were the n-alkane nonacosane and the isopentenyl ester 3-methyl 3-butenyl-(*Z*)-15- tetracosanoate, whereas an unknown compound was commonly overproduced in workers compared to queens (Table 1).

**Table 1:** Compounds with a significant caste-specific difference in European or North American populations (*Mann-Whitney-U tests with Benjamini-Hochberg correction*, *adjusted p* < 0.05). Grey cells indicate a lack of a significant difference in one of the tested populations

Compound name	EU		USA	
	adjusted *p* value	higher expressed in	adjusted p value	higher expressed in
(*Z*)-9-Pentacosene	< 0.001	worker		
Pentacosane	0.036	worker		
3-Methylpentacosane			0.049	worker
3-Methyl 3-butenyl-(*Z*)-11-eicosenoate	0.038	worker		
Unknown 25	< 0.001	queen		
(*Z*)-11-Heptacosene	< 0.001	worker		
(*Z*)-9-Heptacosene			0.023	worker
Unknown 26	0.035	worker	0.032	worker
Heptacosane	0.013	queen		
Unknown 28			0.035	worker
Octacosane			0.020	queen
(*Z*)-11-Nonacosene	0.002	queen		
(*Z*)-7-Nonacosene			0.004	queen
Nonacosane	< 0.001	queen	0.011	queen
3-Methyl 3-butenyl-(*Z*)-15- tetracosanoate	< 0.001	queen	0.002	queen
Unknown 50			0.048	queen
(*Z)*-10-Hentriacontene	0.002	queen	0.049	queen
(*Z*)-9-Hentriacontene	0.032	queen		
Unknown 52			0.009	queen
(*Z*)-12-Tritriacontene	0.040	queen		
Unknown 73			0.004	queen
Unknown 76			0.012	queen

### Body Size Differences

**Fig. 6:**
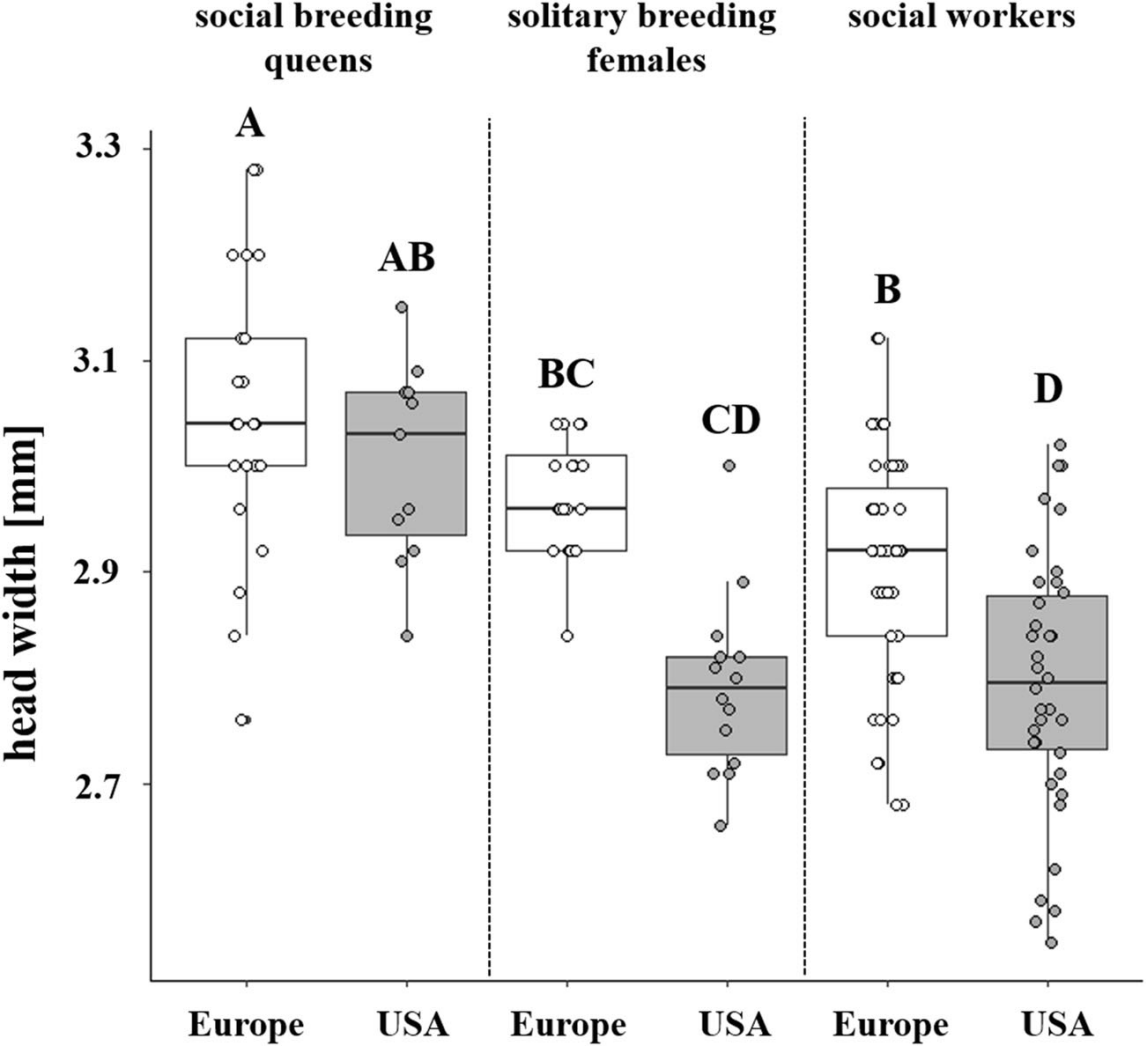
Comparison of the head widths among social breeding queens (EU: n = 22; USA: n = 11), solitary breeding females (EU: n = 20; USA: n = 14) and workers (EU: n = 47; USA: n = 37) between European populations (white) and North American populations (grey). In populations from both continents, social breeding queens were larger than solitary breeding females (EU and USA: *LM* and *Tukey*, *P* < 0.001) and workers (EU and USA: *LM* and *Tukey*, *P* < 0.001), but solitary breeding females exhibited the same size as workers (EU and USA: *LM* and *Tukey*, *P* = 0.99). Beside this, workers from European populations were significantly larger than workers from North America (*LM* and *Tukey*, *P* < 0.001). Boxes represent the median and 25th and 75th percentile, and the overlaying dots represent all calculated data values. Different letters indicate significant differences between the groups

In the European female groups, we found size differences between social breeding queens and solitary breeding females (*linear model*, *F*_*5,146*_ = 18.75, *P* < 0.001, following *Tukey* post-hoc tests, *P* < 0.001; Fig. 6), but not between solitary breeding females and workers (*linear model*, *F*_*5,146*_ = 18.75, *P* < 0.001, following *Tukey* post-hoc tests, *P* = 0.99; Fig. 6). Additionally, the European social breeding queens were larger than their workers (*linear model*, *F*_*5,146*_ = 18.75, *P* < 0.001, following *Tukey* post-hoc tests, *P* < 0.001; Fig. 6). Despite this, size differences between breeding queens and workers was not different among nestmates or non-nestmates (*linear model*, *F*_*3,405*_ = 7.939, *P* = 0.19). Regarding North American populations, social breeding queens were larger than their workers (*linear model*, *F*_*5,146*_ = 18.75, *P* < 0.001, following *Tukey* post-hoc tests, *P* < 0.001; Fig. 6) independent of their nest affiliation (*linear model*, *F*_*3,405*_ = 7.939, *P* = 0.99) and solitary breeding females (*linear model*, F_*5,146*_ = 18.75, *P* < 0.001, following *Tukey* post-hoc tests, *P* < 0.001; Fig. 6), whereas solitary breeding females exhibited the same size as workers (*linear model*, *F*_*5,146*_ = 18.75, *P* < 0.001, following Tukey post-hoc tests, *P* = 0.99; Fig. 6). Comparing females between the European and the North American populations, social breeding queens and solitary breeding queens from both sides of the Atlantic showed the same head widths (*linear model*, *F*_*5,146*_ = 18.75, *P* < 0.001, following *Tukey* post-hoc tests, social breeding: *P* = 0.91, solitary breeding: *P* = 0.13; Fig. 6), whereas workers (*linear model*, *F*_*5,146*_ = 18.75, *P* < 0.001, following *Tukey* post-hoc tests, *P* < 0.001; Fig. 6) were significantly larger in the European populations compared to the North American populations.

## Discussion

In our study, we demonstrate that *H. rubicundus* females from European and North American populations are clearly chemically distinct, independent of their caste or physiological life stage. Additionally, social and solitary populations from both locations exhibit chemical dissimilarities, wherein the European females showed a higher caste-specific and life-stage-specific chemical dissimilarity than the North American ones. Indeed, queens and workers of North American populations could not even be distinguished based on their whole cuticular chemical profile, but only due to the amounts of some single compounds. This may indicate a different importance of inter-caste chemical communication systems between North American and European *H. rubicundus* populations. In Europe, we also found a higher chemical dissimilarity between nest foundresses and breeding females in the social populations compared to the solitary ones. Regarding body sizes, queens of European as well as North American populations were bigger than their workers, whereas the solitary breeding females of both continents were smaller than the social breeding queens and as small as the workers. Overall, the North American females tend to be smaller than the European females.

There was a clear distinction between European and North American populations. We identified more than 50 compounds which were solely found either in the European and North American populations, including different isomers of n-alkenes and macrocyclic lactones, which indicates unique odor profiles that probably arose through chemical ‘drift’ analogous to differentiation through genetic drift for genetic markers. As cuticular hydrocarbons and also macrocyclic lactones are mainly synthesized by the insects themselves and do not have any environmental origin (Blomquist and Bagnères 2010; Holze et al. 2020; Schulz and Hötling 2015), we assume that the differences we found in cuticular chemical profiles are indeed based on genetic differences between populations. Comparable results have already been shown for different species of bumblebees (Bunk et al. 2010; Martin et al. 2010) or stingless bees (Martin et al. 2017), in which different n-alkene isomers separate different species. This raises the question of whether the European and North American *H. rubicundus* females still belong to a single species. However, even if genetic studies demonstrated clearly distinct genetic lineages among both locations, compared to other insect species, divergence could still lie within the range of a single insect species (Soucy and Danforth 2002; Vogler et al. 1993). Nevertheless, the clear chemical distinction of both populations we demonstrated may stimulate more debate on this topic.

Beside this, we hypothesized the existence of caste-specific traits in *H. rubicundus* that were common to European and North American social and solitary populations. Most studies dealing with caste-specific signals in eusocial insects focus on queen signals which regulate worker reproduction; they often indicated conserved signals within a single species or even among several eusocial species of different evolutionary lineages (Oi et al. 2015, 2016; Princen et al. 2020; Van Oystaeyen et al. 2014). This suggests that population ‘dialects’ may not represent caste-specific chemical signals such as queen signals, but may rather influence signals mediating kin or nestmate recognition in social insects. Indeed, there were two common single chemical compounds, the n-alkane nonacosane and the isopentenyl ester 3-methyl 3-butenyl-(Z)-15-tetracosanoate, which are overproduced in queens compared to workers in both populations. If one or both of these compounds may have a function as a queen signal in both populations, it might be an indicator for a conservation of this signal in this species. However, without any behavior assays to test the effect of these compounds on the behavior or physiology of workers, this remains speculative and needs to be further investigated.

Regarding the complete cuticular chemical profile, queens and workers of North American populations seem to be chemically more similar than in Europe and even lack any clear distinction. This raises the question if and how North American and European social colonies differ in their regulation of the reproductive division of labor with European populations relying more on chemical regulation than North American ones. Indeed, it has been hypothesized that chemical regulation of worker reproduction evolved in larger colonies of social insects where behavioral regulation, such as aggressive interactions between the dominant and subordinate females, is limited by a high number of nestmates (Kocher and Grozinger 2011; Ratnieks et al. 2006). Studies on *Polistes* wasps (Toth et al. 2014; Oi et al. 2019) and queenless ants (Cuvillier-Hot et al. 2004; Monnin and Peeters 1999) provide evidence for such an evolutionary transition. It was shown, that dominant and subordinate females of these taxa indeed exhibit differences in their chemical profiles, but these chemical compounds were not sufficient to inhibit the ovarian activity in subordinate females. However, it is rather thought that chemical compounds in these species function as cues to support the effect of a dominant behavior in regulating the reproduction within the colony. These fertility-linked compounds could easily evolve from these cues to real fertility signals or queen pheromones in higher eusocial species (Smith and Liebig 2017). Similar results were recently shown in queens of the bumblebees *Bombus terrestris* and *B. impatiens*, which need direct contact with their workers to inhibit their reproduction, thus queen-produced chemical signals may only function secondarily to augment behavioral cues (Amsalem et al. 2017; Kreuter et al. 2012; Padilla et al. 2016; Starkey et al. 2019). Regarding halictid bees, a recent study using different bioassays to examine the influence of queen-specific chemical compounds on worker behavior and reproductive physiology indicated that the chemical signal, namely macrocyclic lactones, is solely sufficient to function as a queen pheromone, decreasing worker ovarian activation in the obligate eusocial species *L. malachurum* (Steitz and Ayasse 2020). However, even if macrocyclic lactones were shown to be the single common compound class overproduced in queens compared to workers among several halictid bee species (Steitz et al. 2018), we cannot exclude the possibility of the use of behavioral cues to regulate worker reproduction among various halictid bee species. Indeed, as *H. rubicundus* is a facultative eusocial species with a less complex social behavior than e.g. *L. malachurum* or bumblebees, it is also likely that queens use behavioral cues to dominate worker reproduction rather than solely relying on chemical cues. Interestingly, the lower level of caste-specific chemical differences in North American populations suggests such a use of behavioral cues, whereas European females may also use chemical cues due to their clear caste-specific chemical profiles. Further studies, including behavioral assays similar to those described for *L. malachurum* (Steitz and Ayasse 2020) or bumblebees (Kreuter et al. 2012; Padilla et al. 2016), are needed to shed further light on this.

Our results also clearly demonstrate that nest foundresses from social and solitary European populations are chemically more similar than social and solitary breeding females or queens. This leads us to the hypothesis that breeding females of social nests exhibit a queen-specific signal of sociality, as was recently shown in another eusocial halictine bee, *L. pauxillum* (Steitz et al. 2019). Even if it is hypothesized that queen signals evolved from chemicals associated with ovarian development (Oi et al. 2015; Smith and Liebig 2017; Steitz et al. 2018), the occurrence of this social signal in *H. rubicundus* cannot solely be explained by the development of ovaries, but also seems to be affected by social behavior as solitary breeding females change their odor composition to a different extent. Therefore, we would assume that social populations have a stronger or even more honest signal of their fertility than solitary females have. However, it is not appropriate to exclude environmental factors such as climatic conditions which might influence the genetic mechanisms of compound production, since the primary function of cuticular hydrocarbons in insects is to protect against dehydration and other environmental stresses as well as pathogen infection (Blomquist and Bagnères 2010). Indeed, insects are known to adjust their chemical cuticular profile to climatic conditions, not only due to different constant temperatures, but even daily fluctuations (Gibbs and Mousseau 1994; Hadley 1977; Sprenger et al. 2018). Especially for *H. rubicundus,* where the decision to nest either socially or solitarily probably depends on climatic conditions at the nesting site (Yanega 1989; Eickwort et al. 1996; Field et al. 2010), we cannot rule out the idea that the chemical differences we observed among the investigated populations may reflect different environmental or climatic condition at the nesting areas.

In addition to odor differences among populations, castes and breeding status, we also demonstrated that *H. rubicundus* females differ in their body size. Social breeding queens were overall bigger than their workers or solitary breeding queens, a result which we confirm in European and North American populations. North American females were overall smaller than their European counterparts. Soucy (2002) has already shown marked caste dimorphism in North American social *H. rubicundus* in which queens are larger than workers and solitary females. Indeed, bee size is known to be mainly influenced by temperature across several bee species (e.g. Kamm 1974; Radmacher and Strohm 2009; Richards and Packer 1996; Yanega 1989). It is usually assumed that there is an optimum temperature for a given body size such that both lower and higher temperatures cause more stress, resulting in a smaller body size of developing offspring (Soucy 2002). This may be explained by an increase in offspring number with an increasing temperature and consequently a decrease in body size due to a higher number of offspring. On the other side, a lower amount of pollen provisioning due to a lower number of available food sources at decreasing temperatures could also affect body size (Soucy 2002). The lower temperature at locations where *H. rubicundus* nests solitarily may therefore cause a smaller body size in solitary compared with social females. Beside this, the larger body size of European compared with North American populations may indicate that optimal environmental conditions cause a larger body size due to ideal temperature conditions compared to those in North America. Indeed, average temperature values in the summer months are much higher in Logan, Utah, USA than in Hayingen or Bonn, Germany, which may lead to a larger body size in European females than in North American ones. Body size may also influence social behavior, as bigger bees are known to behave more aggressively and to dominate in encounters with smaller bees (Smith and Weller 1989). Indeed, our results indicate that social breeding queens are bigger than their workers in Europe and North America; however, size differences between castes did not differ in North America versus Europe. Therefore, body size differences between castes suggest a similar degree of sociality in North America and Europe and may also be an indication for the use of cues other than chemical signals to maintain reproductive division of labor in *H. rubicundus* nests. Alternatively, the slightly larger worker size of European *H. rubicundus* vs. North American workers might hint at a greater reliance of European queens on chemical in addition to behavioral control of their workers in comparison to those of North America. However, these assumptions are only speculative and further analyses, including especially behavioral experiments on queen-worker behavior and regulation of reproductive division of labor are needed to shed more light on this.

In conclusion, our results clearly show that *H. rubicundus* females from North America and Europe exhibit distinct cuticular chemical profiles, which could be in the range of population dialects or may indicate ongoing speciation. North American and European queens have few common compounds overproduced in comparison to their workers, which might indicate a conservation of a queen signal in this species. However, bioassays testing the effect of these compounds on the behavior and physiology of workers are still needed to shed further light on this issue. As North American females show a lower chemical difference between queens and workers, we assume that the importance of behavioral cues vs. chemical signals in regulating worker reproduction may be different in North American and European populations. A more derived chemical dissimilarity between castes in Europe may indicate the involvement of chemical signals in the regulation of reproductive division of labor, whereas North American populations may also rely on other cues like aggressive behavior of the queen. However, we emphasize the importance of further studies, including behavioral tests, with facultative eusocial halictid bees to shed greater light on the different ways of regulating worker reproduction. This may give new insights into the evolution of social behavior and chemical communication in insects.

## Supplementary Information

ESM 1 (DOCX 525 kb)

## Data Availability

The datasets generated during the current study are available from the corresponding author on request.
