# Supplementary material for: Chemical Variation among Castes, Female Life Stages and Populations of the Facultative Eusocial Sweat Bee Halictus rubicundus (Hymenoptera: Halictidae)
Source: J Chem Ecol. 2021 Mar 31;47(4):406–19. doi: 10.1007/s10886-021-01267-w (PMC8116247; doi:10.1007/s10886-021-01267-w)
Supplement: Supplementary file 1 — (DOCX 525 kb) [file 10886_2021_1267_MOESM1_ESM.docx]

**SUPPLEMENTARY MATERIAL**

Journal of Chemical Ecology

CHEMICAL VARIATION AMONG CASTES, FEMALE LIFE STAGES AND POPULATIONS OF THE FACULTATIVE EUSOCIAL SWEAT BEE *Halictus rubicundus* (HYMENOPTERA: HALICTIDAE)

IRIS STEITZ*^1^, ROBERT J PAXTON^2,3^, STEFAN SCHULZ^4^, MANFRED AYASSE^1^

*^1^ Institute of Evolutionary Ecology and Conservation Genomics, University of Ulm, Ulm, Germany*

*^2^General Zoology, Institute for Biology, Martin Luther University Halle-Wittenberg, Halle (Saale), Germany*

*^3^German Centre for Integrative Biodiversity Research (iDiv) Halle-Jena-Leipzig, Leipzig, Germany*

*^4^Departement of Life Sciences, Institute of Organic Chemistry, TU Braunschweig, Braunschweig, Germany*

**Correspondence: iris.steitz@gmx.net*

**
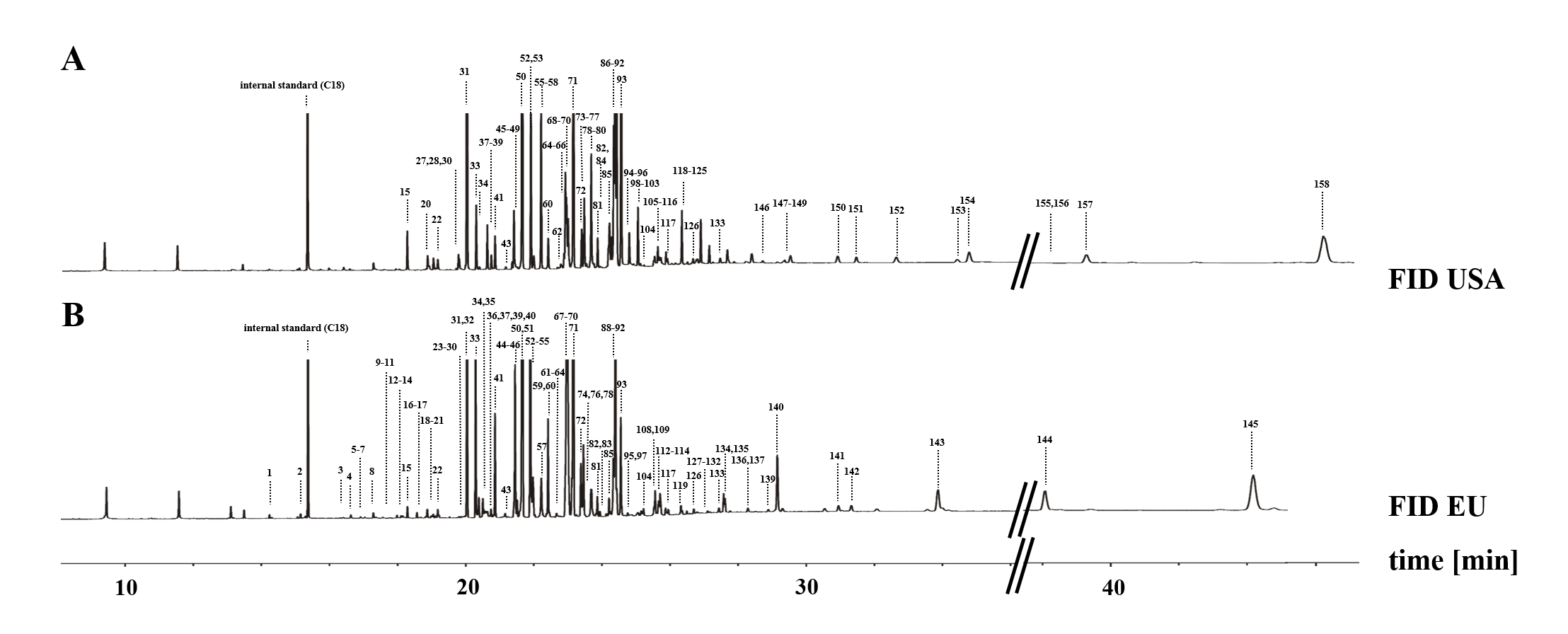
**

**Fig. S1** Comparison of the cuticle surface extract of breeding queens from (A) North American and (B) European *H. rubicundus* populations (for list of compounds see Tab S1)**.**

**Tab. S1** RELATIVE AMOUNTS (%; MEAN ± STANDARD DEVIATION) OF COMPOUNDS FOUND ON THE CUTICLES OF SOCIAL NEST FOUNDRESSES, SOCIAL BREEDING QUEENS, WORKERS, SOLITARY NEST FOUNDRESSES AND SOLITARY BREEDING FEMALES OF *H. RUBICUNDUS* BEES FROM EUROPE AND USA. ASTERISKS INDICATE UNKNOWN DOUBLE BOND POSITIONS

| **Number** | **Compound** | **Retention Index** | **Identification source*** | **Europe** | | | | | **USA** | | | |
| --- | --- | --- | --- | --- | --- | --- | --- | --- | --- | --- | --- | --- |
|  |  |  |  | **social nest foundress** | **worker** | **social breeding queen** | **solitary nest foundress** | **solitary breeding female** | | **worker** | **social breeding queen** | **solitary breeding female** |
| **1** | Heptadecane | 1700 | RC | 0.025 ± 0.031 | 0.048 ± 0.089 | 0.019 ± 0.044 | 0.065 ± 0.063 | 0.039 ± 0.019 | | - | - | - |
| **2** | Ethyl tetradecanoate | 1793 | RC | 0.066 ± 0.103 | 0.033 ± 0.028 | 0.045 ± 0.054 | 0.033 ± 0.046 | 0.025 ± 0.016 | | - | - | - |
| **3** | Nonadecane | 1900 | RC | 0.071 ± 0.049 | 0.195 ± 0.289 | 0.133 ± 0.191 | 0.217 ± 0.182 | 0.194 ± 0.119 | | - | - | - |
| **4** | 16-Hexadecanolide | 1925 | L, RI | 0.004 ± 0.020 | 0.015 ± 0.045 | 0.006 ± 0.015 | 0.008 ± 0.033 | 0.011 ± 0.033 | | - | - | - |
| **5** | Unknown 1 | 1953 |  | 0.026 ± 0.046 | 0.021 ± 0.028 | 0.022 ± 0.035 | 0.031 ± 0.052 | 0.015 ± 0.032 | | - | - | - |
| **6** | Hexadecanoic acid ethyl ester | 1961 | RC | 0.010 ± 0.024 | 0.021 ± 0.029 | 0.024 ± 0.034 | 0.013 ± 0.036 | 0.009 ± 0.016 | | - | - | - |
| **7** | Ethyl hexadecanoate | 1994 | RC | 0.079 ± 0.096 | 0.104 ± 0.088 | 0.187 ± 0.303 | 0.066 ± 0.038 | 0.060 ± 0.036 | | - | - | - |
| **8** | Eicosane | 2000 | RC | 0.030 ± 0.028 | 0.106 ± 0.103 | 0.088 ± 0.099 | 0.144 ± 0.075 | 0.067 ± 0.055 | | - | - | - |
| **9** | Unknown 2 | 2045 |  | 0.007 ± 0.021 | 0.165 ± 0.252 | 0.121 ± 0.230 | 0.014 ± 0.043 | 0.025 ± 0.019 | | - | - | - |
| **10** | Unknown 3 | 2060 |  | - | 0.083 ± 0.132 | 0.052 ± 0.104 | 0.006 ± 0.026 | 0.017 ± 0.017 | | - | - | - |
| **11** | Linoleic acid | 2067 | RC | 0.046 ± 0.049 | 0.055 ± 0.048 | 0.046 ± 0.044 | 0.114 ± 0.074 | 0.032 ± 0.028 | | - | - | - |
| **12** | (*Z*)-9-Heneicosene | 2072 | D, L, RI | 0.183 ± 0.120 | 0.261 ± 0.377 | 0.187 ± 0.276 | 0.296 ± 0.198 | 0.105 ± 0.085 | | - | - | - |
| **13** | (*Z*)-7-Heneicosene | 2079 | D, L, RI | 0.135 ± 0.085 | 0.070 ± 0.063 | 0.066 ± 0.048 | 0.170 ± 0.102 | 0.077 ± 0.091 | | - | - | - |
| **14** | Unknown 4 | 2094 |  | 0.002 ± 0.008 | 0.012 ± 0.030 | 0.006 ± 0.014 | 0.027 ± 0.074 | 0.015 ± 0.012 | | - | - | - |
| **15** | Heneicosane | 2100 | RC | 0.502 ± 0.589 | 0.499 ± 0.686 | 0.487 ± 0.432 | 0.610 ± 0.360 | 2.008 ± 1.172 | | 0.738 ± 0.518 | 0.779 ± 0.517 | 3.004 ± 1.098 |
| **16** | 18-Octadecanolide | 2124 | RC | 0.017 ± 0.037 | 0.033 ± 0.027 | 0.025 ± 0.030 | 0.001 ± 0.003 | 0.090 ± 0.200 | | - | - | - |
| **17** | 18-Octadecenolide* | 2131 | D, L, RI | 0.018 ± 0.031 | 0.038 ± 0.048 | 0.025 ± 0.042 | 0.022 ± 0.049 | 0.049 ± 0.044 | | - | - | - |
| **18** | Ethyl linoleate | 2165 | RC | 0.057 ± 0.045 | 0.121 ± 0.153 | 0.096 ± 0.165 | 0.065 ± 0.058 | 0.179 ± 0.420 | | - | - | - |
| **19** | Ethyl linolenate/ethyl oleate | 2175 | RC | 0.166 ± 0.218 | 0.295 ± 0.292 | 0.635 ± 1.211 | 0.061 ± 0.054 | 0.068 ± 0.104 | | - | - | - |
| **20** | Unknown 5 | 2185 |  | 0.982 ± 0.799 | 0.632 ± 0.523 | 0.631 ± 0.447 | 0.401 ± 0.336 | 0.127 ± 0.164 | | 0.092 ± 0.076 | 0.054 ± 0.070 | 0.212 ± 0.166 |
| **21** | Ethyl octadecanoate | 2193 | RC | 0.016 ± 0.026 | 0.053 ± 0.069 | 0.072 ± 0.092 | 0.052 ± 0.076 | 0.040 ± 0.059 | | - | - | - |
| **22** | Docosane | 2200 | RC | 0.183 ± 0.059 | 0.354 ± 0.443 | 0.229 ± 0.313 | 0.235 ± 0.130 | 0.281 ± 0.112 | | 0.219 ± 0.139 | 0.150 ± 0.088 | 0.380 ± 0.073 |
| **23** | Unknown 6 | 2225 |  | 0.045 ± 0.038 | 0.029 ± 0.037 | 0.012 ± 0.019 | 0.111 ± 0.138 | 0.148 ± 0.126 | | - | - | - |
| **24** | Eicosenoic acid | 2240 | RC | 0.009 ± 0.027 | 0.048 ± 0.051 | 0.050 ± 0.046 | 0.019 ± 0.026 | 0.060 ± 0.042 | | - | - | - |
| **25** | Unknown 7 | 2253 |  | - | 0.022 ± 0.033 | 0.011 ± 0.026 | 0.002 ± 0.006 | 0.020 ± 0.018 | | - | - | - |
| **26** | Tricosene* | 2268 | D, L, RI | 0.011 ± 0.053 | 0.356 ± 1.489 | 0.525 ± 1.199 | 0.005 ± 0.016 | 0.108 ± 0.234 | | - | - | - |
| **27** | (*Z*)-11-Tricosene | 2273 | D, L, RI | 0.075 ± 0.113 | 0.319 ± 0.378 | 0.194 ± 0.322 | 0.091 ± 0.073 | 0.562 ± 0.517 | | 0.318 ± 0.333 | 0.711 ± 0.720 | 0.179 ± 0.251 |
| **28** | (*Z*)-9-Tricosene | 2279 | D, L, RI | 0.076 ± 0.115 | 0.063 ± 0.061 | 0.108 ± 0.126 | 0.068 ± 0.065 | 0.034 ± 0.029 | | 0.035 ± 0.030 | 0.031 ± 0.010 | 0.056 ± 0.057 |
| **29** | (*Z*)-7-Tricosene | 2284 | D, L, RI | 0.064 ± 0.053 | 0.066 ± 0.035 | 0.055 ± 0.061 | 0.078 ± 0.085 | 0.053 ± 0.039 | | - | - | - |
| **30** | (*Z*)-5-Tricosene | 2294 | D, L, RI | 0.053 ± 0.037 | 0.022 ± 0.022 | 0.032 ± 0.024 | 0.134 ± 0.069 | 0.103 ± 0.054 | | 0.053 ± 0.037 | 0.041 ± 0.015 | 0.062 ± 0.044 |
| **31** | Tricosane | 2300 | RC | 11.542 ± 4.544 | 5.319 ± 3.811 | 7.201 ± 3.329 | 9.784 ± 3.684 | 11.806 ± 4.814 | | 12.335 ± 4.623 | 8.417 ± 2.657 | 15.675 ± 2.777 |
| **32** | Unknown 8 | 2320 |  | 0.143 ± 0.397 | 0.041 ± 0.113 | 0.056 ± 0.205 | 0.010 ± 0.021 | 0.077 ± 0.118 | | - | - | - |
| **33** | 20-Eicosanolide | 2332 | RC | 2.312 ± 3.339 | 4.332 ± 5.468 | 4.076 ± 4.254 | 1.085 ± 2.367 | 10.174 ± 6.950 | | 0.901 ± 0.693 | 0.630 ± 0.422 | 0.940 ± 0.617 |
| **34** | (*Z*)-Eicos-9-en-20-olide | 2342 | D, L, RI | 0.490 ± 0.682 | 0.543 ± 0.640 | 0.582 ± 0.474 | 0.165 ± 0.090 | 1.865 ± 1.244 | | 0.058 ± 0.046 | 0.038 ± 0.031 | 0.118 ± 0.093 |
| **35** | (*Z*)-Eicos-11-en-20-olide | 2349 | D, L, RI | 0.578 ± 0.720 | 0.034 ± 0.041 | 0.029 ± 0.033 | 1.014 ± 0.620 | 0.492 ± 0.420 | | - | - | - |
| **36** | Unknown 9 | 2355 |  | 0.985 ± 0.526 | 0.055 ± 0.070 | 0.289 ± 0.450 | 0.559 ± 0.331 | 0.296 ± 0.245 | | - | - | - |
| **37** | Unknown 10 | 2362 |  | 0.376 ± 0.188 | 0.151 ± 0.117 | 0.167 ± 0.135 | 0.061 ± 0.098 | 0.081 ± 0.121 | | 0.012 ± 0.018 | 0.003 ± 0.007 | 0.056 ± 0.036 |
| **38** | Unknown 11 | 2370 |  | - | - | - | - | - | | 0.209 ± 0.481 | 0.077 ± 0.053 | 0.419 ± 0.207 |
| **39** | Unknown 12 | 2385 |  | 0.959 ± 0.734 | 0.324 ± 0.426 | 0.427 ± 0.384 | 0.785 ± 0.527 | 0.305 ± 0.362 | | 0.125 ± 0.101 | 0.104 ± 0.105 | 0.400 ± 0.180 |
| **40** | Ethyl eicosanoate | 2395 | RC | 0.094 ± 0.183 | 0.095 ± 0.454 | 0.183 ± 0.729 | 0.033 ± 0.033 | 0.019 ± 0.013 | | - | - | - |
| **41** | Tetracosane | 2400 | RC | 0.370 ± 0.212 | 1.420 ± 1.573 | 0.692 ± 0.871 | 0.351 ± 0.134 | 0.338 ± 0.145 | | 0.625 ± 0.281 | 0.513 ± 0.280 | 0.560 ± 0.096 |
| **42** | Docosenoic acid | 2429 | RC | 0.063 ± 0.062 | 0.127 ± 0.093 | 0.125 ± 0.077 | 0.178 ± 0.110 | 0.189 ± 0.070 | | - | - | - |
| **43** | Unknown 13 | 2437 |  | 0.020 ± 0.042 | 0.023 ± 0.026 | 0.024 ± 0.026 | 0.055 ± 0.058 | 0.079 ± 0.095 | | 0.040 ± 0.023 | 0.023 ± 0.014 | 0.117 ± 0.100 |
| **44** | (*Z*)-11-Pentacosene | 2449 | D, L, RI | 0.860 ± 0.841 | 2.831 ± 2.982 | 3.616 ± 2.650 | 0.306 ± 0.216 | 1.426 ± 1.422 | | - | - | - |
| **45** | (*Z*)-10-Pentacosene | 2463 | D, L, RI | - | - | - | - | - | | 0.048 ± 0.105 | 0.007 ± 0.017 | 0.087 ± 0.065 |
| **46** | (*Z*)-9-Pentacosene | 2470 | D, L, RI | 0.829 ± 0.830 | 0.803 ± 0.864 | 0.229 ± 0.251 | 0.387 ± 0.166 | 1.409 ± 1.552 | | 1.200 ± 0.860 | 2.337 ± 1.517 | 0.162 ± 0.081 |
| **47** | Pentacosene 1* | 2478 | D, L, RI | - | - | - | - | - | | 0.036 ± 0.023 | 0.023 ± 0.015 | 0.207 ± 0.069 |
| **48** | Pentacosene 2* | 2481 | D, L, RI | - | - | - | - | - | | 0.057 ± 0.021 | 0.038 ± 0.021 | 0.076 ± 0.056 |
| **49** | 3-Methylpentacosane | 2494 | L, RI | - | - | - | - | - | | 0.171 ± 0.162 | 0.059 ± 0.025 | 0.101 ± 0.036 |
| **50** | Pentacosane | 2500 | RC | 11.678 ± 4.792 | 17.675 ± 7.730 | 11.666 ± 2.844 | 9.176 ± 3.341 | 9.088 ± 3.855 | | 15.857 ± 3.239 | 14.026 ± 2.315 | 14.953 ± 1.800 |
| **51** | Unknown 15 | 2515 |  | - | 0.059 ± 0.101 | 0.050 ± 0.107 | 0.028 ± 0.047 | 0.022 ± 0.027 | | - | - | - |
| **52** | 22-Docosanolide | 2533 | RC | 4.247 ± 4.396 | 7.102 ± 7.565 | 6.224 ± 4.864 | 7.135 ± 9.815 | 11.582 ± 7.757 | | 2.120 ± 1.661 | 1.472 ± 0.897 | 2.031 ± 1.525 |
| **53** | (*Z*)-Docos-11-en-22-olide | 2543 | D, L, RI | 1.062 ± 1.423 | 1.534 ± 1.796 | 1.422 ± 1.112 | 1.349 ± 2.210 | 3.985 ± 2.588 | | 0.262 ± 0.201 | 0.169 ± 0.110 | 0.415 ± 0.349 |
| **54** | (*Z*)-Docos-13-en-22-olide | 2550 | D, L, RI | 0.014 ± 0.027 | 0.062 ± 0.061 | 0.053 ± 0.038 | 0.065 ± 0.076 | 0.060 ± 0.041 | | - | - | - |
| **55** | Unknown 16 | 2563 |  | 0.120 ± 0.147 | 0.160 ± 0.180 | 0.196 ± 0.134 | 0.008 ± 0.030 | 0.051 ± 0.095 | | 0.011 ± 0.020 | 0.014 ± 0.027 | 0.055 ± 0.047 |
| **56** | Unknown 17 | 2569 |  | - | - | - | - | - | | 0.084 ± 0.061 | 0.097 ± 0.049 | 0.009 ± 0.017 |
| **57** | Unknown 18 | 2573 |  | 0.100 ± 0.079 | 0.049 ± 0.084 | 0.025 ± 0.033 | 0.119 ± 0.073 | 0.265 ± 0.361 | | 0.746 ± 2.051 | 0.023 ± 0.023 | 0.093 ± 0.037 |
| **58** | Unknown 19 | 2584 |  | - | - | - | - | - | | 0.023 ± 0.014 | 0.015 ± 0.015 | 0.012 ± 0.018 |
| **59** | Ethyl docosanoate | 2595 | RC | 0.011 ± 0.057 | 0.030 ± 0.041 | 0.015 ± 0.024 | 0.158 ± 0.193 | 0.043 ± 0.102 | | - | - | - |
| **60** | Hexacosane | 2600 | RC | 0.463 ± 0.139 | 1.559 ± 1.613 | 1.047 ± 1.197 | 0.358 ± 0.186 | 0.294 ± 0.157 | | 0.616 ± 0.137 | 0.756 ± 0.489 | 0.744 ± 0.273 |
| **61** | Unknown 20 | 2623 |  | 0.028 ± 0.040 | 0.047 ± 0.041 | 0.040 ± 0.052 | 0.086 ± 0.088 | 0.013 ± 0.016 | | - | - | - |
| **62** | 3-Methyl 3-butenyl-(*Z*)-11-eicosenoate | 2634 | RC | 0.081 ± 0.063 | 0.085 ± 0.054 | 0.059 ± 0.039 | 0.124 ± 0.095 | 0.071 ± 0.039 | | 0.031 ± 0.020 | 0.022 ± 0.015 | 0.058 ± 0.034 |
| **63** | Unknown 21 | 2641 |  | 0.023 ± 0.044 | 0.039 ± 0.054 | 0.017 ± 0.029 | 0.063 ± 0.092 | 0.102 ± 0.054 | | - | - | - |
| **64** | Unknown 22 | 2648 |  | 0.171 ± 0.239 | 0.067 ± 0.068 | 0.044 ± 0.039 | 0.332 ± 0.302 | 0.058 ± 0.052 | | 0.137 ± 0.094 | 0.128 ± 0.052 | 0.093 ± 0.127 |
| **65** | Unknown 23 | 2656 |  | - | - | - | - | - | | 0.039 ± 0.031 | 0.011 ± 0.013 | 0.102 ± 0.050 |
| **66** | Unknown 24 | 2661 |  | - | - | - | - | - | | 0.114 ± 0.109 | 0.031 ± 0.039 | 0.030 ± 0.034 |
| **67** | Unknown 25 | 2664 |  | 0.717 ± 1.190 | 1.550 ± 4.170 | 4.670 ± 4.992 | 0.616 ± 0.452 | 0.227 ± 0.114 | | - | - | - |
| **68** | (*Z*)-11-Heptacosene | 2668 | D, L, RI | 6.649 ± 2.790 | 12.310 ± 8.890 | 1.082 ± 1.607 | 5.955 ± 3.143 | 0.069 ± 0.078 | | 2.954 ± 1.317 | 2.669 ± 1.032 | 0.446 ± 0.387 |
| **69** | (*Z*)-9-Heptacosene | 2678 | D, L, RI | 2.860 ± 1.779 | 1.014 ± 2.034 | 0.123 ± 0.153 | 3.156 ± 1.816 | 0.056 ± 0.050 | | 0.779 ± 0.353 | 0.405 ± 0.135 | 2.211 ± 0.478 |
| **70** | Unknown 26 | 2690 |  | 0.367 ± 0.330 | 0.191 ± 0.111 | 0.104 ± 0.114 | 0.019 ± 0.050 | 0.036 ± 0.034 | | 0.162 ± 0.174 | 0.011 ± 0.014 | 0.269 ± 0.117 |
| **71** | Heptacosane | 2700 | RC | 9.898 ± 3.050 | 10.484 ± 4.156 | 14.182 ± 3.074 | 11.819 ± 4.642 | 6.046 ± 2.662 | | 13.206 ± 3.273 | 12.996 ± 1.287 | 13.610 ± 2.566 |
| **72** | 24-Tetracosanolide | 2731 | RC | 1.145 ± 1.075 | 1.097 ± 0.799 | 0.903 ± 0.556 | 1.838 ± 2.355 | 0.977 ± 0.625 | | 0.456 ± 0.309 | 0.314 ± 0.174 | 0.474 ± 0.294 |
| **73** | 24-Tetracosenolide 1* | 2743 | D, L, RI | - | - | - | - | - | | 0.766 ± 0.582 | 0.466 ± 0.289 | 0.963 ± 0.813 |
| **74** | (*Z*)-Tetracos-11-en-24-olide | 2752 | D, L, RI | 1.945 ± 2.247 | 2.062 ± 2.376 | 1.586 ± 1.342 | 3.616 ± 4.792 | 4.222 ± 3.138 | | 0.049 ± 0.064 | 0.038 ± 0.020 | 0.030 ± 0.031 |
| **75** | 24-Tetracosenolide 2* | 2757 | D, L, RI | - | - | - | - | - | | 0.001 ± 0.004 | 0.009 ± 0.019 | 0.008 ± 0.019 |
| **76** | (*Z*)-Tetracos-13-en-24-olide | 2763 | D, L, RI | 0.152 ± 0.160 | 0.150 ± 0.188 | 0.121 ± 0.117 | 0.069 ± 0.037 | 0.305 ± 0.538 | | 0.022 ± 0.023 | 0.025 ± 0.040 | 0.045 ± 0.048 |
| **77** | 24-Tetracosenolide 3* | 2768 | D, L, RI | - | - | - | - | - | | 0.102 ± 0.062 | 0.120 ± 0.024 | 0.072 ± 0.060 |
| **78** | (*Z*)-Tetracos-15-en-24-olide | 2774 | D, L, RI | 0.878 ± 0.416 | 0.849 ± 0.412 | 0.943 ± 0.356 | 0.921 ± 0.314 | 0.531 ± 0.400 | | 0.958 ± 1.416 | 1.016 ± 0.432 | 0.200 ± 0.065 |
| **79** | Unknown 27 | 2784 |  | - | - | - | - | - | | 0.111 ± 0.056 | 0.128 ± 0.024 | 0.224 ± 0.210 |
| **80** | Unknown 28 | 2791 |  | - | - | - | - | - | | 0.053 ± 0.045 | 0.006 ± 0.014 | 0.067 ± 0.065 |
| **81** | Octacosane | 2800 | RC | 0.233 ± 0.073 | 0.634 ± 0.651 | 0.691 ± 0.574 | 0.262 ± 0.118 | 0.279 ± 0.126 | | 0.619 ± 0.169 | 1.055 ± 0.521 | 0.773 ± 0.301 |
| **82** | Unknown 29 | 2811 |  | 0.152 ± 0.112 | 0.413 ± 1.173 | 0.255 ± 0.223 | 0.761 ± 0.608 | 0.403 ± 0.922 | | 0.103 ± 0.074 | 0.111 ± 0.089 | 0.471 ± 0.218 |
| **83** | Unknown 30 | 2827 |  | 0.021 ± 0.045 | 0.137 ± 0.582 | 0.037 ± 0.064 | 0.008 ± 0.018 | 0.018 ± 0.061 | | - | - | - |
| **84** | Unknown 31 | 2834 |  | - | - | - | - | - | | 0.017 ± 0.022 | 0.008 ± 0.011 | 0.069 ± 0.046 |
| **85** | 3-Methyl 3-butenyl-(*Z*)-13-docosenoate | 2838 | RC | 0.055 ± 0.055 | 0.175 ± 0.508 | 0.462 ± 1.152 | 0.035 ± 0.032 | 0.044 ± 0.028 | | 0.026 ± 0.023 | 0.036 ± 0.028 | 0.044 ± 0.042 |
| **86** | Unknown 32 | 2844 |  | - | - | - | - | - | | 0.589 ± 0.380 | 0.923 ± 0.719 | - |
| **87** | Unknown 33 | 2848 |  | - | - | - | - | - | | 0.171 ± 0.376 | 0.295 ± 0.357 | 0.467 ± 1.095 |
| **88** | Unknown 34 | 2850 |  | 1.355 ± 1.108 | 0.132 ± 0.174 | 0.283 ± 0.305 | 1.391 ± 1.023 | 0.081 ± 0.102 | | 0.798 ± 0.530 | 1.163 ± 1.088 | 1.714 ± 0.796 |
| **89** | Unknown 35 | 2857 |  | 1.241 ± 1.011 | 0.121 ± 0.123 | 0.158 ± 0.213 | 1.870 ± 1.088 | 0.586 ± 1.756 | | 0.530 ± 0.301 | 0.428 ± 0.357 | 1.642 ± 0.722 |
| **90** | (*Z*)-11-Nonacosene | 2867 | D, L, RI | 3.887 ± 4.306 | 0.818 ± 1.897 | 3.623 ± 3.339 | 1.263 ± 0.669 | 0.198 ± 0.124 | | 2.023 ± 0.698 | 2.550 ± 0.666 | 0.902 ± 0.276 |
| **91** | (*Z*)-9-Nonacosene | 2873 | D, L, RI | 7.343 ± 2.703 | 5.688 ± 4.343 | 2.612 ± 2.702 | 6.433 ± 2.211 | 0.267 ± 0.186 | | 7.135 ± 2.050 | 8.159 ± 1.432 | 5.265 ± 1.317 |
| **92** | (*Z*)-7-Nonacosene | 2880 | D, L, RI | 2.256 ± 1.686 | 0.973 ± 1.911 | 0.369 ± 0.419 | 2.442 ± 0.870 | 0.124 ± 0.129 | | 4.171 ± 2.058 | 6.679 ± 1.160 | 0.844 ± 0.327 |
| **93** | Nonacosane | 2900 | RC | 2.409 ± 0.861 | 2.174 ± 1.481 | 5.855 ± 2.306 | 3.588 ± 1.842 | 4.868 ± 2.245 | | 10.119 ± 3.747 | 16.231 ± 4.758 | 8.759 ± 1.914 |
| **94** | Unknown 36 | 2920 |  | - | - | - | - | - | | 0.050 ± 0.039 | 0.041 ± 0.018 | 0.084 ± 0.053 |
| **95** | Unknown 37 | 2930 |  | 0.126 ± 0.106 | 0.120 ± 0.266 | 0.104 ± 0.096 | 0.148 ± 0.137 | 0.147 ± 0.065 | | 0.053 ± 0.226 | 0.009 ± 0.012 | 0.062 ± 0.047 |
| **96** | Unknown 38 | 2937 |  | - | - | - | - | - | | 0.165 ± 0.590 | 0.007 ± 0.011 | 0.003 ± 0.012 |
| **97** | Unknown 39 | 2939 |  | 0.166 ± 0.097 | 0.133 ± 0.221 | 0.194 ± 0.168 | 0.151 ± 0.091 | 0.201 ± 0.196 | | - | - | - |
| **98** | Unknown 40 | 2942 |  | - | - | - | - | - | | 0.033 ± 0.044 | 0.018 ± 0.025 | 0.168 ± 0.128 |
| **99** | 26-Hexacosenolide 1* | 2947 | D, L, RI | - | - | - | - | - | | 0.048 ± 0.036 | 0.070 ± 0.022 | 0.070 ± 0.089 |
| **100** | 26-Hexacosenolide 2* | 2953 | D, L, RI | - | - | - | - | - | | 0.075 ± 0.103 | 0.070 ± 0.062 | 0.084 ± 0.045 |
| **101** | Unknown 41 | 2962 |  | - | - | - | - | - | | 0.031 ± 0.027 | 0.039 ± 0.035 | 0.123 ± 0.065 |
| **102** | Unknown 42 | 2976 |  | - | - | - | - | - | | 0.467 ± 0.986 | 0.260 ± 0.086 | 0.210 ± 0.067 |
| **103** | Unknown 43 | 2985 |  | - | - | - | - | - | | 0.103 ± 0.118 | 0.068 ± 0.028 | 0.506 ± 0.205 |
| **104** | Triacontane | 3000 | RC | 0.082 ± 0.067 | 0.178 ± 0.195 | 0.154 ± 0.166 | 0.228 ± 0.148 | 0.193 ± 0.449 | | 0.061 ± 0.057 | 0.202 ± 0.331 | 0.338 ± 0.173 |
| **105** | Unknown 44 | 3011 |  | - | - | - | - | - | | 0.044 ± 0.065 | 0.016 ± 0.015 | 0.437 ± 0.493 |
| **106** | Unknown 45 | 3035 |  | - | - | - | - | - | | 0.019 ± 0.025 | 0.016 ± 0.014 | 0.156 ± 0.093 |
| **107** | Unknown 46 | 3039 |  | - | - | - | - | - | | 0.009 ± 0.023 | 0.003 ± 0.009 | 0.031 ± 0.049 |
| **108** | 3-Methyl 3-butenyl-(*Z*)-15- tetracosanoate | 3043 | RC | 1.299 ± 1.078 | 0.147 ± 0.186 | 1.589 ± 1.781 | 0.063 ± 0.113 | 0.066 ± 0.082 | | 0.077 ± 0.047 | 0.273 ± 0.181 | 0.347 ± 0.442 |
| **109** | Unknown 47 | 3050 |  | 2.145 ± 1.286 | 0.212 ± 0.405 | 0.873 ± 1.968 | 0.541 ± 0.351 | 0.223 ± 0.152 | | 0.070 ± 0.052 | 0.052 ± 0.054 | 0.401 ± 0.630 |
| **110** | Unknown 48 | 3057 |  | - | - | - | - | - | | 0.039 ± 0.036 | 0.042 ± 0.047 | 1.720 ± 1.245 |
| **111** | Unknown 49 | 3064 |  | - | - | - | - | - | | 0.140 ± 0.233 | 0.030 ± 0.041 | 0.497 ± 0.151 |
| **112** | Unknown 50 | 3069 |  | 0.758 ± 0.551 | 0.088 ± 0.174 | 0.202 ± 0.435 | 0.883 ± 0.431 | 0.011 ± 0.033 | | 0.060 ± 0.057 | 0.120 ± 0.057 | 0.721 ± 0.380 |
| **113** | (*Z)*-10-Hentriacontene | 3075 | D, L, RI | 0.792 ± 0.616 | 0.260 ± 0.419 | 2.865 ± 3.487 | 0.283 ± 0.108 | 0.667 ± 0.373 | | 0.302 ± 0.182 | 0.423 ± 0.124 | 1.243 ± 1.012 |
| **114** | (*Z*)-9-Hentriacontene | 3082 | D, L, RI | 1.954 ± 1.438 | 1.073 ± 1.702 | 2.530 ± 2.056 | 1.200 ± 0.881 | 1.842 ± 0.935 | | 0.102 ± 0.075 | 0.079 ± 0.063 | 0.354 ± 0.622 |
| **115** | Unknown 51 | 3090 |  | - | - | - | - | - | | 0.038 ± 0.051 | 0.058 ± 0.046 | 0.232 ± 0.224 |
| **116** | Unknown 52 | 3093 |  | - | - | - | - | - | | 0.008 ± 0.022 | 0.066 ± 0.047 | 0.010 ± 0.033 |
| **117** | Hentriacontane | 3100 | RC | 0.338 ± 0.301 | 0.232 ± 0.175 | 0.309 ± 0.209 | 0.244 ± 0.114 | 0.497 ± 0.294 | | 0.185 ± 0.271 | 0.175 ± 0.224 | 0.487 ± 0.212 |
| **118** | Unknown 53 | 3106 |  | - | - | - | - | - | | 0.581 ± 0.797 | 0.174 ± 0.051 | 0.458 ± 0.318 |
| **119** | Unknown 54 | 3113 |  | 0.188 ± 0.220 | 0.105 ± 0.084 | 0.136 ± 0.108 | 0.327 ± 0.235 | 0.484 ± 0.469 | | 0.061 ± 0.083 | 0.058 ± 0.034 | 0.133 ± 0.117 |
| **120** | Unknown 55 | 3137 |  | - | - | - | - | - | | 0.074 ± 0.098 | 0.004 ± 0.008 | 0.098 ± 0.088 |
| **121** | Unknown 56 | 3143 |  | - | - | - | - | - | | 0.049 ± 0.103 | 0.014 ± 0.021 | 0.070 ± 0.108 |
| **122** | Unknown 57 | 3149 |  | - | - | - | - | - | | 0.023 ± 0.024 | 0.044 ± 0.032 | 0.093 ± 0.096 |
| **123** | Unknown 58 | 3163 |  | - | - | - | - | - | | 0.056 ± 0.069 | 0.043 ± 0.054 | 0.157 ± 0.159 |
| **124** | Unknown 59 | 3173 |  | - | - | - | - | - | | 0.409 ± 1.067 | 0.082 ± 0.060 | 0.080 ± 0.107 |
| **125** | Unknown 60 | 3178 |  | - | - | - | - | - | | 0.052 ± 0.046 | 0.044 ± 0.021 | 0.396 ± 0.266 |
| **126** | Dotriacontane | 3200 | RC | 0.162 ± 0.455 | 0.102 ± 0.119 | 0.079 ± 0.091 | 0.122 ± 0.078 | 0.207 ± 0.327 | | 0.066 ± 0.052 | 0.110 ± 0.134 | 1.303 ± 1.014 |
| **127** | Unknown 61 | 3233 |  | 0.216 ± 0.688 | 0.101 ± 0.125 | 0.127 ± 0.136 | 0.378 ± 0.339 | 0.126 ± 0.094 | | - | - | - |
| **128** | Unknown 62 | 3245 |  | 0.618 ± 0.554 | 0.145 ± 0.154 | 1.649 ± 2.289 | 0.435 ± 0.226 | 1.525 ± 1.250 | | - | - | - |
| **129** | Unknown 63 | 3253 |  | 0.398 ± 0.359 | 0.123 ± 0.134 | 0.219 ± 0.283 | 0.819 ± 0.636 | 0.517 ± 0.347 | | - | - | - |
| **130** | (*Z*)-12-Tritriacontene | 3268 | D, L, RI | 0.353 ± 0.753 | 0.109 ± 0.122 | 1.447 ± 2.298 | 0.383 ± 0.376 | 3.631 ± 1.759 | | - | - | - |
| **131** | Tritriacontene 1* | 3276 | D, L, RI | 0.110 ± 0.165 | 0.122 ± 0.214 | 0.127 ± 0.205 | 0.277 ± 0.244 | 1.356 ± 0.977 | | - | - | - |
| **132** | Tritriacontene 2* | 3286 | D, L, RI | 0.111 ± 0.226 | 0.048 ± 0.093 | 0.061 ± 0.066 | 0.117 ± 0.107 | 0.259 ± 0.233 | | - | - | - |
| **133** | Tritriacontane | 3300 | RC | 0.164 ± 0.197 | 0.125 ± 0.120 | 0.118 ± 0.065 | 0.700 ± 0.313 | 0.553 ± 0.382 | | 0.196 ± 0.359 | 0.104 ± 0.085 | 0.093 ± 0.049 |
| **134** | Stigmasterol |  | RC | 0.108 ± 0.141 | 0.189 ± 0.202 | 0.176 ± 0.137 | 0.339 ± 0.111 | 0.265 ± 0.223 | | - | - | - |
| **135** | Fucosterol |  | RC | 0.065 ± 0.172 | 0.098 ± 0.107 | 0.122 ± 0.096 | 0.268 ± 0.281 | 0.276 ± 0.182 | | - | - | - |
| **136** | Unknown 64 |  |  | 0.249 ± 0.265 | 0.207 ± 0.247 | 0.187 ± 0.132 | 0.261 ± 0.231 | 0.377 ± 0.583 | | - | - | - |
| **137** | Unknown 65 |  |  | 0.225 ± 0.438 | 0.136 ± 0.148 | 0.320 ± 0.400 | 0.519 ± 0.475 | 1.213 ± 0.553 | | - | - | - |
| **138** | Unknown 66 |  |  | 0.131 ± 0.502 | 0.062 ± 0.115 | 0.108 ± 0.352 | 0.511 ± 0.503 | 0.759 ± 0.435 | | - | - | - |
| **139** | Unknown 67 |  |  | 0.272 ± 0.275 | 0.250 ± 0.482 | 0.774 ± 1.914 | 0.293 ± 0.154 | 1.201 ± 0.855 | | - | - | - |
| **140** | Hexodecyl oleate |  | RC | 0.214 ± 0.404 | 0.117 ± 0.108 | 0.113 ± 0.096 | 0.270 ± 0.172 | 0.312 ± 0.278 | | - | - | - |
| **141** | Unknown 68 |  |  | 0.279 ± 0.235 | 0.089 ± 0.114 | 0.079 ± 0.135 | 0.576 ± 0.363 | 0.284 ± 0.254 | | - | - | - |
| **142** | Unknown 69 |  |  | 0.068 ± 0.151 | 0.056 ± 0.077 | 0.067 ± 0.098 | 0.065 ± 0.056 | 0.061 ± 0.051 | | - | - | - |
| **143** | Wax type ester 1 |  | L, RI | 1.168 ± 0.441 | 1.402 ± 1.764 | 0.803 ± 0.332 | 2.314 ± 0.892 | 1.596 ± 0.708 | | - | - | - |
| **144** | Wax type ester 2 |  | L, RI | 1.336 ± 0.732 | 1.277 ± 0.538 | 0.853 ± 0.362 | 2.160 ± 1.029 | 1.341 ± 0.826 | | - | - | - |
| **145** | Wax type ester 3 |  | L, RI | 1.765 ± 1.583 | 3.016 ± 1.270 | 2.815 ± 1.160 | 2.136 ± 1.176 | 1.660 ± 1.834 | | - | - | - |
| **146** | Unknown 70 |  |  | - | - | - | - | - | | 0.315 ± 0.439 | 0.281 ± 0.134 | 0.339 ± 0.459 |
| **147** | Fatty acid cytronellyl ester 1 |  | L, RI | - | - | - | - | - | | 0.082 ± 0.073 | 0.097 ± 0.071 | 0.235 ± 0.233 |
| **148** | Fatty acid cytronellyl ester 2 |  | L, RI | - | - | - | - | - | | 0.080 ± 0.105 | 0.132 ± 0.147 | 0.130 ± 0.126 |
| **149** | Fatty acid octadecenoic ester 1 |  | L, RI | - | - | - | - | - | | 0.972 ± 3.181 | 0.275 ± 0.111 | 0.362 ± 0.190 |
| **150** | Unknown 71 |  |  | - | - | - | - | - | | 0.043 ± 0.060 | 0.043 ± 0.058 | 0.402 ± 0.369 |
| **151** | Unknown 72 |  |  | - | - | - | - | - | | 0.983 ± 1.180 | 1.601 ± 0.832 | 0.784 ± 1.018 |
| **152** | Unknown 73 |  |  | - | - | - | - | - | | 0.206 ± 0.523 | 1.190 ± 1.119 | 0.086 ± 0.094 |
| **153** | Unknown 74 |  |  | - | - | - | - | - | | 0.180 ± 0.272 | 0.126 ± 0.108 | 0.052 ± 0.063 |
| **154** | Unknown 75 |  |  | - | - | - | - | - | | 1.417 ± 3.578 | 0.575 ± 0.258 | 0.675 ± 0.222 |
| **155** | Unknown 76 |  |  | - | - | - | - | - | | 0.048 ± 0.091 | 0.175 ± 0.164 | 0.006 ± 0.012 |
| **156** | Fatty acid octadecenoic ester 2 |  | L, RI | - | - | - | - | - | | 0.570 ± 0.655 | 0.301 ± 0.056 | 0.509 ± 0.180 |
| **157** | Fatty acid octadecenoic ester 3 |  | L, RI | - | - | - | - | - | | 2.747 ± 1.084 | 2.201 ± 0.480 | 1.374 ± 0.480 |
| **158** | Unknown 77 |  |  | - | - | - | - | - | | 5.384 ± 2.824 | 3.548 ± 1.695 | 2.908 ± 1.108 |

* Identification source: RC = reference compound, L = NIST library, RI = retention index, D = derivatization

**Tab. S2** *BRAY-CURTIS-DISSIMILARITIES OF COMPOUNDS (SIMPER ANALYSES, >90 % CONTRIBUTION TO THE SEPARATION OF GROUPS) AMONG ALL GROUP COMPARISONS OF SOCIAL NEST FOUNDRESSES, SOCIAL BREEDING QUEENS, WORKERS, SOLITARY NEST FOUNDRESSES AND SOLITARY BREEDING FEMALES OF H. RUBICUNDUS BEES FROM EUROPE AND USA*

| **Compound** | **EU vs. US females** | | | **US females** | | **EU females** | | | | |
| --- | --- | --- | --- | --- | --- | --- | --- | --- | --- | --- |
|  | **workers** | **social breeding queens** | **solitary breeding females** | **social breeding queens vs. solitary breeding females** | **workers vs. social breeding queens** | **social vs. solitary nest foundresses** | **social vs. solitary breeding females** | **workers vs. social breeding queens** | **social nest foundresses vs. breeding queens** | **solitary nest foundresses vs. breeding females** |
| Ethyl tetradecanoate | 0.00 | 0.00 | 0.00 | 0.00 | 0.00 | 0.14 | 0.00 | 0.00 | 0.20 | 0.00 |
| Nonadecane | 0.29 | 0.24 | 0.33 | 0.00 | 0.00 | 0.17 | 0.19 | 0.22 | 0.24 | 0.00 |
| Ethyl hexadecanoate | 0.25 | 0.28 | 0.18 | 0.00 | 0.00 | 0.00 | 0.17 | 0.19 | 0.00 | 0.00 |
| Eicosane | 0.24 | 0.21 | 0.19 | 0.00 | 0.00 | 0.18 | 0.00 | 0.00 | 0.00 | 0.00 |
| Unknown 2 | 0.25 | 0.29 | 0.00 | 0.00 | 0.00 | 0.00 | 0.16 | 0.24 | 0.21 | 0.00 |
| Unknown 3 | 0.00 | 0.00 | 0.00 | 0.00 | 0.00 | 0.00 | 0.00 | 0.17 | 0.00 | 0.00 |
| Linoleic acid | 0.18 | 0.00 | 0.00 | 0.00 | 0.00 | 0.14 | 0.00 | 0.00 | 0.00 | 0.00 |
| (*Z*)-9-Heneicosene | 0.29 | 1.00 | 0.23 | 0.69 | 0.00 | 0.16 | 0.20 | 0.31 | 0.58 | 0.19 |
| (*Z*)-7-Heneicosene | 0.20 | 1.72 | 0.19 | 0.00 | 0.00 | 0.00 | 0.00 | 0.00 | 0.00 | 0.14 |
| Heneicosane | 0.35 | 0.31 | 0.40 | 0.71 | 0.27 | 0.25 | 0.59 | 0.30 | 0.18 | 0.48 |
| 18-Octadecanolide | 0.00 | 0.00 | 0.00 | 0.00 | 0.00 | 0.00 | 0.00 | 0.00 | 0.00 | 0.16 |
| Ethyl linoleate | 0.22 | 0.19 | 0.23 | 0.00 | 0.00 | 0.00 | 0.20 | 0.20 | 0.37 | 0.16 |
| Ethyl linolenate/ethyl oleate | 0.41 | 0.50 | 0.00 | 0.00 | 0.00 | 0.19 | 0.36 | 0.35 | 0.15 | 0.00 |
| Unknown 5 | 0.41 | 0.32 | 0.22 | 0.23 | 0.15 | 0.40 | 0.41 | 0.00 | 0.18 | 0.30 |
| Ethyl octadecanoate | 0.00 | 0.17 | 0.00 | 0.00 | 0.00 | 0.00 | 0.00 | 0.16 | 0.00 | 0.00 |
| Docosane | 0.24 | 0.18 | 0.00 | 0.20 | 0.12 | 0.00 | 0.21 | 0.27 | 0.21 | 0.00 |
| Unknown 6 | 0.00 | 0.00 | 0.27 | 0.00 | 0.00 | 0.00 | 0.23 | 0.00 | 0.00 | 0.15 |
| Tricosene* | 0.19 | 0.34 | 0.00 | 0.00 | 0.00 | 0.00 | 0.35 | 0.41 | 0.21 | 0.14 |
| (*Z*)-11-Tricosene | 0.29 | 0.71 | 0.36 | 0.00 | 0.36 | 0.00 | 0.37 | 0.30 | 0.55 | 0.33 |
| (*Z*)-9-Tricosene | 0.00 | 0.00 | 0.00 | 0.00 | 0.00 | 0.15 | 0.16 | 0.18 | 0.00 | 0.00 |
| (*Z*)-7-Tricosene | 0.21 | 0.00 | 0.00 | 0.00 | 0.00 | 0.14 | 0.00 | 0.00 | 0.00 | 0.00 |
| Tricosane | 1.22 | 0.54 | 0.62 | 0.89 | 0.70 | 0.59 | 0.79 | 0.76 | 0.33 | 0.62 |
| Unknown 8 | 0.00 | 0.00 | 0.00 | 0.00 | 0.11 | 0.14 | 0.17 | 0.00 | 0.52 | 0.15 |
| 20-Eicosanolide | 0.93 | 0.96 | 1.64 | 0.28 | 0.31 | 0.79 | 1.35 | 1.07 | 0.96 | 1.86 |
| (*Z*)-Eicos-9-en-20-olide | 0.38 | 0.46 | 0.77 | 0.16 | 0.00 | 0.24 | 0.56 | 0.35 | 0.35 | 0.69 |
| (*Z*)-Eicos-11-en-20-olide | 0.00 | 0.00 | 0.50 | 0.00 | 0.00 | 0.48 | 0.42 | 0.00 | 0.40 | 0.31 |
| Unknown 9 | 0.15 | 0.22 | 0.39 | 0.00 | 0.00 | 0.27 | 0.30 | 0.28 | 0.21 | 0.23 |
| Unknown 10 | 0.26 | 0.32 | 0.00 | 0.16 | 0.00 | 0.36 | 0.23 | 0.16 | 0.40 | 0.16 |
| Unknown 11 | 0.26 | 0.29 | 0.49 | 0.30 | 0.18 | 0.00 | 0.00 | 0.00 | 0.00 | 0.00 |
| Unknown 12 | 0.26 | 0.30 | 0.22 | 0.28 | 0.16 | 0.31 | 0.28 | 0.32 | 0.18 | 0.34 |
| Ethyl eicosanoate | 0.00 | 0.00 | 0.00 | 0.00 | 0.00 | 0.16 | 0.16 | 0.20 | 0.20 | 0.00 |
| Tetracosane | 0.38 | 0.28 | 0.00 | 0.00 | 0.17 | 0.17 | 0.26 | 0.49 | 0.00 | 0.00 |
| Docosenoic acid | 0.29 | 0.28 | 0.33 | 0.00 | 0.00 | 0.17 | 0.00 | 0.00 | 0.00 | 0.00 |
| Unknown 13 | 0.00 | 0.00 | 0.00 | 0.16 | 0.00 | 0.00 | 0.00 | 0.00 | 0.00 | 0.14 |
| (*Z*)-11-Pentacosene | 1.25 | 0.44 | 0.77 | 0.00 | 0.00 | 0.34 | 0.85 | 0.83 | 0.21 | 0.55 |
| (*Z*)-10-Pentacosene | 0.00 | 0.00 | 0.21 | 0.16 | 0.00 | 0.00 | 0.00 | 0.00 | 0.00 | 0.00 |
| (*Z*)-9-Pentacosene | 0.39 | 0.17 | 0.54 | 0.00 | 0.55 | 0.27 | 0.55 | 0.37 | 0.19 | 0.50 |
| Pentacosene 1* | 0.00 | 0.00 | 0.35 | 0.23 | 0.00 | 0.00 | 0.00 | 0.00 | 0.00 | 0.00 |
| Pentacosene 2* | 0.20 | 0.00 | 0.18 | 0.00 | 0.00 | 0.00 | 0.00 | 0.00 | 0.00 | 0.00 |
| 3-Methylpentacosane | 0.32 | 0.46 | 0.24 | 0.28 | 0.16 | 0.00 | 0.00 | 0.00 | 0.00 | 0.00 |
| Pentacosane | 0.74 | 0.41 | 0.75 | 0.26 | 0.39 | 0.69 | 0.58 | 0.85 | 0.18 | 0.56 |
| 22-Docosanolide | 1.07 | 1.04 | 1.55 | 0.42 | 0.45 | 1.21 | 1.18 | 1.04 | 0.55 | 1.56 |
| (*Z*)-Docos-11-en-22-olide | 0.56 | 0.61 | 1.04 | 0.26 | 0.16 | 0.59 | 0.77 | 0.54 | 0.51 | 0.98 |
| (*Z*)-Docos-13-en-22-olide | 0.17 | 0.17 | 0.00 | 0.00 | 0.00 | 0.14 | 0.00 | 0.00 | 0.92 | 0.00 |
| Unknown 16 | 0.24 | 1.56 | 0.00 | 0.00 | 0.00 | 0.21 | 0.26 | 0.15 | 0.18 | 0.00 |
| Unknown 17 | 0.22 | 0.73 | 0.00 | 0.00 | 0.11 | 0.00 | 0.00 | 0.00 | 0.00 | 0.00 |
| Unknown 18 | 0.34 | 0.00 | 0.00 | 0.15 | 0.32 | 0.00 | 0.28 | 0.22 | 0.19 | 0.16 |
| Ethyl docosanoate | 0.00 | 0.00 | 0.00 | 0.00 | 0.00 | 0.23 | 0.00 | 0.00 | 0.00 | 0.19 |
| Hexacosane | 0.37 | 0.26 | 0.26 | 0.15 | 0.14 | 0.00 | 0.33 | 0.43 | 0.17 | 0.15 |
| Unknown 20 | 0.16 | 0.00 | 0.00 | 0.00 | 0.00 | 0.16 | 0.00 | 0.00 | 0.00 | 0.15 |
| Unknown 21 | 0.00 | 0.00 | 0.24 | 0.00 | 0.00 | 0.14 | 0.19 | 0.00 | 0.00 | 0.17 |
| Unknown 22 | 0.17 | 0.00 | 0.00 | 0.15 | 0.00 | 0.25 | 0.00 | 0.00 | 1.11 | 0.25 |
| Unknown 23 | 0.00 | 0.00 | 0.23 | 0.00 | 0.11 | 0.00 | 0.00 | 0.00 | 0.00 | 0.00 |
| Unknown 24 | 0.24 | 0.00 | 0.00 | 0.00 | 0.19 | 0.00 | 0.00 | 0.00 | 0.00 | 0.00 |
| Unknown 25 | 0.44 | 0.19 | 0.36 | 0.00 | 0.00 | 0.40 | 1.13 | 1.48 | 0.27 | 0.28 |
| (*Z*)-11-Heptacosene | 1.63 | 0.69 | 0.34 | 0.00 | 0.34 | 0.59 | 0.55 | 2.09 | 0.84 | 1.65 |
| (*Z*)-9-Heptacosene | 0.49 | 1.33 | 1.00 | 0.85 | 0.23 | 0.47 | 0.00 | 0.50 | 1.19 | 1.15 |
| Unknown 26 | 0.22 | 0.18 | 0.29 | 0.36 | 0.25 | 0.39 | 0.00 | 0.20 | 0.16 | 0.00 |
| Heptacosane | 0.62 | 0.30 | 1.00 | 0.26 | 0.31 | 0.59 | 1.08 | 0.64 | 0.24 | 0.88 |
| 24-Tetracosanolide | 0.37 | 0.34 | 0.30 | 0.17 | 0.19 | 0.52 | 0.29 | 0.32 | 0.25 | 0.51 |
| 24-Tetracosenolide 1* | 0.56 | 0.49 | 0.92 | 0.30 | 0.28 | 0.89 | 0.85 | 0.58 | 0.16 | 1.01 |
| (*Z*)-Tetracos-11-en-24-olide | 0.16 | 0.00 | 0.00 | 0.21 | 0.00 | 0.00 | 0.00 | 0.00 | 0.00 | 0.00 |
| (*Z*)-Tetracos-13-en-24-olide | 0.21 | 0.19 | 0.24 | 0.00 | 0.00 | 0.00 | 0.22 | 0.20 | 0.19 | 0.20 |
| 24-Tetracosenolide 3* | 0.25 | 0.30 | 0.18 | 0.15 | 0.00 | 0.00 | 0.00 | 0.00 | 0.00 | 0.00 |
| (*Z*)-Tetracos-15-en-24-olide | 0.37 | 0.20 | 0.20 | 0.44 | 0.40 | 0.17 | 0.27 | 0.21 | 0.31 | 0.25 |
| Unknown 27 | 0.28 | 0.76 | 0.29 | 0.16 | 0.00 | 0.00 | 0.00 | 0.00 | 0.00 | 0.00 |
| Unknown 28 | 0.16 | 0.00 | 0.00 | 0.74 | 0.15 | 0.00 | 0.00 | 0.00 | 0.00 | 0.00 |
| Octacosane | 0.27 | 0.28 | 0.28 | 0.18 | 0.21 | 0.00 | 0.23 | 0.28 | 0.58 | 0.00 |
| Unknown 29 | 0.21 | 0.55 | 0.24 | 0.29 | 0.12 | 0.36 | 0.20 | 0.22 | 0.00 | 0.34 |
| Unknown 30 | 0.00 | 0.00 | 0.00 | 0.00 | 0.00 | 0.00 | 0.00 | 0.17 | 0.55 | 0.00 |
| Unknown 31 | 0.00 | 0.00 | 0.18 | 0.43 | 0.00 | 0.00 | 0.00 | 0.00 | 0.00 | 0.00 |
| 3-Methyl 3-butenyl-(*Z*)-13-docosenoate | 0.18 | 0.25 | 0.00 | 0.00 | 0.00 | 0.00 | 0.22 | 0.28 | 0.54 | 0.00 |
| Unknown 32 | 0.63 | 0.31 | 0.00 | 0.00 | 0.28 | 0.00 | 0.00 | 0.00 | 0.00 | 0.00 |
| Unknown 33 | 0.17 | 0.16 | 0.21 | 0.20 | 0.35 | 0.00 | 0.00 | 0.00 | 0.00 | 0.00 |
| Unknown 34 | 0.53 | 0.31 | 0.82 | 0.52 | 0.49 | 0.42 | 0.26 | 0.27 | 0.59 | 0.68 |
| Unknown 35 | 0.37 | 0.20 | 0.90 | 0.55 | 0.22 | 0.45 | 0.36 | 0.20 | 0.15 | 0.94 |
| (*Z*)-11-Nonacosene | 0.78 | 1.43 | 0.41 | 0.43 | 0.25 | 0.63 | 1.07 | 1.05 | 0.87 | 0.52 |
| (*Z*)-9-Nonacosene | 0.79 | 0.85 | 1.41 | 0.00 | 0.34 | 0.44 | 0.84 | 1.13 | 0.37 | 1.55 |
| (*Z*)-7-Nonacosene | 1.17 | 0.26 | 0.48 | 0.34 | 0.58 | 0.41 | 0.28 | 0.44 | 0.22 | 0.96 |
| Nonacosane | 1.50 | 1.36 | 0.67 | 0.87 | 0.83 | 0.41 | 0.48 | 0.87 | 0.00 | 0.49 |
| Unknown 36 | 0.17 | 0.22 | 0.21 | 0.00 | 0.00 | 0.00 | 0.00 | 0.00 | 0.00 | 0.00 |
| Unknown 37 | 0.21 | 0.33 | 0.00 | 0.00 | 0.00 | 0.16 | 0.00 | 0.17 | 0.16 | 0.00 |
| Unknown 38 | 0.00 | 0.00 | 0.00 | 0.25 | 0.14 | 0.00 | 0.00 | 0.00 | 0.00 | 0.00 |
| Unknown 39 | 0.25 | 0.42 | 0.31 | 0.00 | 0.00 | 0.00 | 0.17 | 0.20 | 0.83 | 0.14 |
| Unknown 40 | 0.00 | 0.00 | 0.27 | 0.00 | 0.11 | 0.00 | 0.00 | 0.00 | 0.00 | 0.00 |
| 26-Hexacosenolide 1* | 0.16 | 0.20 | 0.00 | 0.15 | 0.00 | 0.00 | 0.00 | 0.00 | 0.00 | 0.00 |
| 26-Hexacosenolide 2* | 0.17 | 0.42 | 0.20 | 0.00 | 0.16 | 0.00 | 0.00 | 0.00 | 0.00 | 0.00 |
| Unknown 41 | 0.00 | 0.00 | 0.26 | 0.36 | 0.00 | 0.00 | 0.00 | 0.00 | 0.00 | 0.00 |
| Unknown 42 | 0.43 | 0.21 | 0.35 | 0.39 | 0.25 | 0.00 | 0.00 | 0.00 | 0.00 | 0.00 |
| Unknown 43 | 0.26 | 0.18 | 0.54 | 0.22 | 0.00 | 0.00 | 0.00 | 0.00 | 0.00 | 0.00 |
| Triacontane | 0.17 | 0.19 | 0.25 | 0.26 | 0.18 | 0.18 | 0.16 | 0.17 | 0.75 | 0.18 |
| Unknown 44 | 0.00 | 0.00 | 0.44 | 0.86 | 0.12 | 0.00 | 0.00 | 0.00 | 0.00 | 0.00 |
| Unknown 45 | 0.00 | 0.00 | 0.29 | 0.26 | 0.00 | 0.00 | 0.00 | 0.00 | 0.00 | 0.00 |
| 3-Methyl 3-butenyl-(*Z*)-15- tetracosanoate | 0.18 | 0.56 | 0.32 | 0.38 | 0.22 | 0.70 | 0.69 | 0.68 | 0.18 | 0.16 |
| Unknown 47 | 0.19 | 0.25 | 0.36 | 0.35 | 0.14 | 0.57 | 0.37 | 0.41 | 0.51 | 0.25 |
| Unknown 48 | 0.00 | 0.00 | 0.91 | 0.48 | 0.11 | 0.00 | 0.00 | 0.00 | 0.00 | 0.00 |
| Unknown 49 | 0.24 | 0.00 | 0.54 | 0.18 | 0.20 | 0.00 | 0.00 | 0.00 | 0.00 | 0.00 |
| Unknown 50 | 0.18 | 0.26 | 0.60 | 0.42 | 0.14 | 0.24 | 0.21 | 0.25 | 0.15 | 0.67 |
| (*Z*)-10-Hentriacontene | 0.25 | 0.76 | 0.44 | 0.53 | 0.14 | 0.28 | 0.75 | 0.93 | 1.39 | 0.24 |
| (*Z*)-9-Hentriacontene | 0.51 | 0.31 | 0.79 | 0.48 | 0.13 | 0.42 | 0.52 | 0.73 | 1.06 | 0.36 |
| Unknown 51 | 0.00 | 0.19 | 0.33 | 0.20 | 0.13 | 0.00 | 0.00 | 0.00 | 0.00 | 0.00 |
| Unknown 52 | 0.00 | 0.35 | 0.00 | 0.20 | 0.18 | 0.00 | 0.00 | 0.00 | 0.00 | 0.00 |
| Hentriacontane | 0.19 | 0.21 | 0.00 | 0.29 | 0.18 | 0.14 | 0.21 | 0.18 | 0.58 | 0.20 |
| Unknown 53 | 0.57 | 0.23 | 0.50 | 0.00 | 0.25 | 0.00 | 0.00 | 0.00 | 0.00 | 0.00 |
| Unknown 54 | 0.00 | 0.00 | 0.31 | 0.16 | 0.11 | 0.20 | 0.29 | 0.00 | 0.20 | 0.26 |
| Unknown 55 | 0.16 | 0.00 | 0.20 | 0.00 | 0.16 | 0.00 | 0.00 | 0.00 | 0.00 | 0.00 |
| Unknown 56 | 0.00 | 0.00 | 0.00 | 0.21 | 0.12 | 0.00 | 0.00 | 0.00 | 0.00 | 0.00 |
| Unknown 57 | 0.00 | 0.00 | 0.19 | 0.17 | 0.11 | 0.00 | 0.00 | 0.00 | 0.00 | 0.00 |
| Unknown 58 | 0.16 | 0.00 | 0.25 | 0.34 | 0.14 | 0.00 | 0.00 | 0.00 | 0.00 | 0.00 |
| Unknown 59 | 0.32 | 0.17 | 0.00 | 0.21 | 0.23 | 0.00 | 0.00 | 0.00 | 0.00 | 0.00 |
| Unknown 60 | 0.17 | 0.43 | 0.43 | 0.20 | 0.00 | 0.00 | 0.00 | 0.00 | 0.00 | 0.00 |
| Dotriacontane | 0.00 | 0.00 | 0.55 | 0.63 | 0.12 | 0.21 | 0.18 | 0.15 | 0.21 | 0.15 |
| Unknown 61 | 0.23 | 0.79 | 0.26 | 0.00 | 0.00 | 0.29 | 0.00 | 0.16 | 0.59 | 0.21 |
| Unknown 62 | 0.28 | 0.33 | 0.86 | 0.00 | 0.00 | 0.21 | 0.67 | 0.62 | 0.28 | 0.46 |
| Unknown 63 | 0.25 | 0.34 | 0.51 | 0.00 | 0.00 | 0.33 | 0.31 | 0.20 | 0.00 | 0.28 |
| (*Z*)-12-Tritriacontene | 0.22 | 0.19 | 1.42 | 1.37 | 0.00 | 0.23 | 0.96 | 0.61 | 0.75 | 0.97 |
| Tritriacontene 1* | 0.21 | 0.20 | 0.79 | 0.00 | 0.00 | 0.26 | 0.68 | 0.23 | 0.19 | 0.56 |
| Tritriacontene 2* | 0.00 | 0.18 | 0.35 | 0.00 | 0.00 | 0.21 | 0.25 | 0.16 | 0.17 | 0.20 |
| Tritriacontane | 0.16 | 0.00 | 0.32 | 0.00 | 0.16 | 0.37 | 0.30 | 0.00 | 0.00 | 0.21 |
| Stigmasterol | 0.33 | 0.33 | 0.36 | 0.00 | 0.00 | 0.26 | 0.16 | 0.15 | 0.31 | 0.15 |
| Fucosterol | 0.22 | 0.26 | 0.39 | 0.00 | 0.00 | 0.28 | 0.18 | 0.16 | 0.27 | 0.23 |
| Unknown 64 | 0.35 | 0.39 | 0.37 | 0.00 | 0.00 | 0.24 | 0.23 | 0.14 | 0.32 | 0.29 |
| Unknown 65 | 0.28 | 0.56 | 0.82 | 0.00 | 0.00 | 0.37 | 0.51 | 0.24 | 0.19 | 0.40 |
| Unknown 66 | 0.00 | 0.00 | 0.65 | 0.00 | 0.00 | 0.46 | 0.58 | 0.18 | 0.28 | 0.29 |
| Unknown 67 | 0.35 | 0.16 | 0.81 | 0.00 | 0.00 | 0.16 | 0.49 | 0.33 | 0.29 | 0.41 |
| Hexodecyl oleate | 0.27 | 0.26 | 0.41 | 0.00 | 0.00 | 0.21 | 0.19 | 0.00 | 0.72 | 0.14 |
| Unknown 68 | 0.21 | 0.17 | 0.39 | 0.00 | 0.00 | 0.28 | 0.28 | 0.16 | 0.16 | 0.22 |
| Unknown 69 | 0.15 | 0.48 | 0.00 | 0.00 | 0.00 | 0.15 | 0.00 | 0.31 | 0.38 | 0.00 |
| Wax type ester 1 | 0.94 | 0.75 | 0.96 | 0.00 | 0.00 | 0.35 | 0.32 | 0.27 | 0.25 | 0.28 |
| Wax type ester 2 | 0.96 | 1.37 | 0.87 | 0.51 | 0.00 | 0.33 | 0.28 | 0.24 | 0.53 | 0.35 |
| Wax type ester 3 | 1.47 | 0.81 | 0.94 | 0.82 | 0.00 | 0.44 | 0.47 | 0.34 | 0.76 | 0.34 |
| Unknown 70 | 0.42 | 0.23 | 0.39 | 0.00 | 0.19 | 0.00 | 0.00 | 0.00 | 0.00 | 0.00 |
| Fatty acid cytronellyl ester 1 | 0.22 | 0.26 | 0.33 | 0.00 | 0.14 | 0.00 | 0.00 | 0.00 | 0.00 | 0.00 |
| Fatty acid cytronellyl ester 2 | 0.18 | 0.43 | 0.26 | 0.36 | 0.19 | 0.00 | 0.00 | 0.00 | 0.00 | 0.00 |
| Fatty acid octadecenoic ester 1 | 0.53 | 1.02 | 0.45 | 0.47 | 0.29 | 0.00 | 0.00 | 0.00 | 0.00 | 0.00 |
| Unknown 71 | 0.00 | 0.00 | 0.42 | 0.61 | 0.14 | 0.00 | 0.00 | 0.00 | 0.00 | 0.00 |
| Unknown 72 | 0.76 | 0.79 | 0.61 | 0.18 | 0.46 | 0.00 | 0.00 | 0.00 | 0.00 | 0.00 |
| Unknown 73 | 0.27 | 0.26 | 0.18 | 0.00 | 0.62 | 0.00 | 0.00 | 0.00 | 0.00 | 0.00 |
| Unknown 74 | 0.29 | 0.62 | 0.00 | 0.27 | 0.20 | 0.00 | 0.00 | 0.00 | 0.00 | 0.00 |
| Unknown 75 | 0.79 | 0.31 | 0.63 | 0.00 | 0.28 | 0.00 | 0.00 | 0.00 | 0.00 | 0.00 |
| Unknown 76 | 0.00 | 0.19 | 0.00 | 0.00 | 0.23 | 0.00 | 0.00 | 0.00 | 0.00 | 0.00 |
| Fatty acid octadecenoic ester 2 | 0.60 | 1.23 | 0.55 | 0.36 | 0.17 | 0.00 | 0.00 | 0.00 | 0.00 | 0.00 |
| Fatty acid octadecenoic ester 3 | 1.40 | 1.53 | 0.90 | 0.21 | 0.29 | 0.00 | 0.00 | 0.00 | 0.00 | 0.00 |
| Unknown 77 | 1.58 | 1.33 | 0.00 | 1.92 | 0.60 | 0.00 | 0.00 | 0.00 | 0.00 | 0.59 |
